# Cancelling Flash Illusory Line Motion by Cancelling the Attentional Gradient and a Consideration of Consciousness

**DOI:** 10.3390/vision3010003

**Published:** 2019-01-10

**Authors:** Katie McGuire, Amanda Pinny, Jeff P. Hamm

**Affiliations:** School of Psychology, The University of Auckland, Auckland 1023, New Zealand

**Keywords:** illusory line motion, visual attention, consciousness

## Abstract

Illusory line motion (ILM) refers to the perception of motion in a line that is, in fact, presented in full at one time. One form of this illusion (_flash_ILM) occurs when the line is presented between two objects following a brief luminance change in one of them and _flash_ILM is thought to result from exogenous attention being captured by the flash. Exogenous attention fades with increasing delays, which predicts that _flash_ILM should show a similar temporal pattern. Exogenous attention appears to follow _flash_ILM to become more or less equally distributed along the line.The current study examines _flash_ILM in order to test these predictions derived from the attentional explanation for _flash_ILM and the results were consistent with them. The discussion then concludes with an exploratory analysis approach concerning states of consciousness and decision making and suggests a possible role for attention.

## 1. Introduction

Illusory line motion (ILM) refers to the perception of motion in a bar that is presented all at once. ILM can occur under a number of different conditions. ILM can occur when the bar appears next to a sole existing stimulus [1,2], when it is referred to as polarised gamma motion (PGM). ILM can also occur when the bar is presented and joins two objects that differ in some attribute, such as colour or shape, and the bar matches one of the objects on this attribute [3,4,5,6], in which case it is referred to as transformational apparent motion (TAM). Finally, ILM occurs when the bar appears between two existing stimuli subsequent to one of the items undergoing a brief luminance change [6,7,8,9,10,11], when the illusion is referred to as _flash_ILM.

The illusory motion that arises in _flash_ILM, TAM, and PGM can be quantified in a single value referred to as ILM_area_. This metric is obtained by presenting the bar in real leftward or rightward motion at various speeds while also inducing leftward or rightward illusory motion. Participants respond by indicating the direction in which the bar appears to move, scoring rightward responses as +1 and leftward as −1, and averaging these “percept scores” over a number of presentations. This scoring results in a measure akin to a guess corrected accuracy. The percept scores are plotted as a series over the real motion separately for left and right side inducers, and the area between these series can be used to quantify a participant’s illusion (see [11] for more information). This measure is highly stable, and participants who show a large illusion in one condition will show a large illusion in another condition provided both conditions reflect the same underlying illusion. For example, TAM can arise when the boxes and bar match based upon colour or based upon size, and the ILM_area_ measures for these two illusions are highly correlated [6] as both are thought to reflect the tracking of objects over time [3,4]. The ILM_area_ from the TAM illusion, however, is not correlated with the ILM_area_ from _flash_ILM [6,12], despite variations on the _flash_ILM paradigm also showing high test-retest reliability on this measure [6,10,11,12]. 

PGM was rediscovered by Hikosaka et al. [13] in the same year that _flash_ILM was introduced by the same lab [14]. At that time both were suggested to arise as a result of the workings of visual exogenous attention and how it is thought to be distributed as a gradient [15] centred at either the single inducing item (PGM) or the flash (_flash_ILM). Subsequent work, however, has indicated that PGM appears to be generated in early visual cortex through the spreading of sub-threshold activation [16] while _flash_ILM has been associated with exogenous visual attention through multiple lines of evidence: ILM_area_ and the costs plus benefits from an exogenous cuing task have been shown to be correlated [8]; fMRI activation patterns during the viewing of _flash_ILM include both motion and attention related areas [9]; a patient population with deficits in attention [7] shows a reduced ILM_area_ relative to controls. In addition, participants’ ILM_area_ from PGM and _flash_ILM displays are not correlated with each other nor are ILM_area_ measures between PGM and TAM. This pattern is consistent with the separate explanations for _flash_ILM, TAM, and PGM [12].

The current set of studies are specifically concerned with _flash_ILM and the proposed relationship it has with the gradient of exogenous attention and is not concerned with either TAM or PGM. To reiterate, _flash_ILM occurs when one of two initial boxes flashes briefly prior to the onset or offset [10,11] of a bar that joins the two boxes. The illusory motion signal is thought to arise because exogenous attention is asymmetrically distributed to be focused upon the flashed box, and so is asymmetrically focused at one end of the bar upon its appearance. It is also possible to generate _flash_ILM in an existing bar by changing its colour upon the offset of the flash [7,12], which is also explained due to the asymmetric distribution of exogenous attention. When the colour change paradigm needs to be distinguished from the onset/offset bar paradigm we will refer to the former as _paint_ILM and the latter as _flash_ILM, but the former will be used only when it is necessary to highlight the paradigm difference, otherwise the term _flash_ILM will be used as _paint_ILM and _flash_ILM produce high test-retest reliability in the ILM_area_ measure and neither produces any logical challenges to the common attention based explanation current in the literature. In short, _paint_ILM is used to refer to a particular methodological protocol used to generate the _flash_ILM phenomenon.

The costs plus benefits of non-informative exogenous cues have a limited duration post-cue and may be followed by inhibition of return (IOR; [17] for review). It follows that _flash_ILM should also diminish as the presentation of the bar is delayed relative to the flash. Because IOR may be a process that is revealed as exogenous attention fades over time [18] and does not appear to result in ILM [19], there is no reason to expect the illusion to reverse direction unless attention were to shift focus to the terminus location. A reversal of the illusion is indicated when ILM_area_ is negative [10,11], which occurs because ILM_area_ is always calculated as the area under the percept curve following a left inducer minus the area under the percept curve following a right inducer. The distribution of attention has also been shown to be influenced subsequent to _flash_ILM, with attention redistributing itself to encompass both the flashed and non-flashed locations [20]. A similar finding is reported in experiment three by Hsieh et al. [21].

If attention is redistributed to be less asymmetric following an instance of _flash_ILM [20] then a subsequent change should produce weaker _flash_ILM. Because _flash_ILM can be examined using the _paint_ILM paradigm [12,22], if an existing bar changes colour twice following a flash, _flash_ILM should be reduced for the second colour change as the first instance of _flash_ILM should have disrupted the asymmetry of the attentional gradient through redistribution. Hamm and Klein’s [20] results found no significant difference in the cuing effect at either end of the bar following _flash_ILM, suggesting that _flash_ILM may even drop below detectable levels. It could be, however, that _flash_ILM is more sensitive to small asymmetries than the costs and benefits calculated from response time data, but even so the prediction is clear that _flash_ILM should be reduced if it is associated with the asymmetrical gradient of exogenous attention.

Two experiments employing the _paint_ILM paradigm are presented here testing these predictions that arise from the attentional gradient explanation for _flash_ILM. Experiment one tracks ILM_area_ over increasing flash-bar SOA in order to determine whether ILM_area_ reduces, as predicted by a fading gradient over time. Experiment two measures ILM_area_ at the two shortest SOA intervals when the colour change is the only colour change. In addition, ILM_area_ is measured at the second of these intervals when it is preceded by an initial colour change upon flash offset. If _flash_ILM is related to the distribution of exogenous attention, then the illusion should be observable at both intervals during the single colour change conditions but it should be reduced, possibly below detectable levels, when it is the second of the two colour changes. If ILM_area_ does not reduce with increasing SOA, or it does not reduce following an initial instance of _flash_ILM, then such findings would be difficult to reconcile under the attentional gradient explanation for _flash_ILM and so these experiments pose a challenge to the current explanation.

These hypotheses will be examined using null hypothesis significance testing (NHST), which is an assessment of the accuracy of the predictions derived from the null hypothesis. The null is deemed to be inaccurate when the observed data are deemed improbable to occur when one starts with the assertion that the null is true. The assessment of improbability is reflected in the standard *p* value. Failure to reject the null, it must be remembered, is not a claim that the null is true, only that any differences between the observed data and the null prediction could reasonably have arisen simply due to measurement variability of a common population mean. In other words, the null could be false despite non-significance and there could be additional effects. However, if that is the case, then those effects are presumed to be of insufficient magnitude to be detected under the current conditions. Therefore, their existence can only be hypothetical speculations, currently unsupported by the data. Increasing sample sizes is one way to reduce the range of hypothetical-non-detectable effects, as this increases statistical power, but the range can never be reduced to zero. The flip side is also the case, in that there is a known probability of rejecting the null hypothesis in error, and so significance is never a guarantee that a proposed effect is true, only that the data is unlikely to have been obtained if there is, in fact, no effect at all. We will discuss effects as having been demonstrated should the null hypothesis be rejected. However, when the null has not been rejected, we will present these as situations where any possible differences have fallen below detectable levels.

Bayesian probabilities can be determined from analysis of variance and correlations [23] or from *t*-tests [24]. The Bayesian probability, denoted as pH0|D, is an assessment of the strength of the evidence for or against the null hypothesis, and this can be converted back to the Bayes Factor (BF) odds ratio simply by BF = pH0|D/(1 − pH0|D). When used in combination, rather than as competing mutually exclusive methods, these two approaches provide clearer guidance on the theoretical interpretations of experimental evidence. While NHST p values tend to be described as either significant or non-significant, with some occasionally using phrases such as “marginally significant” (which really should be phrased “marginally non-significant” if it is to be used at all), Bayesian pH0|D values tend to be described using more graded language. We will employ the descriptions suggested by Raftery [25], with the addition of the equivocal description for the range (0.475–0.525) [8,12] if necessary. The Bayesian analysis may be viewed as guidance towards future investigations for hypothetically possible effects that may exist but are below detectable levels.

## 2. Experiment 1

### 2.1. Methods

#### 2.1.1. Participants

Twelve males and twelve females (21 right handed and three left handed as determined by [26]) between the ages of 20 and 31 (mean age 22.5 years) gave voluntary written informed consent to participate in this study. All reported normal or corrected-to-normal vision. The procedures were approved by The University of Auckland Human Participant’s Ethics Committee (approval code number 2006/065).

#### 2.1.2. Apparatus

Stimuli were presented on a 14-inch colour monitor with a screen resolution of 640 × 480 pixels with the background colour set to grey and a screen refresh rate of 60 Hz. Stimulus presentation was controlled by a 486 computer running DOS as recommended by [27]. The millisecond timing routines are described in [28], with synchronization of the timing with the screen refresh cycle [29].

#### 2.1.3. Stimuli

**Figure 1:**
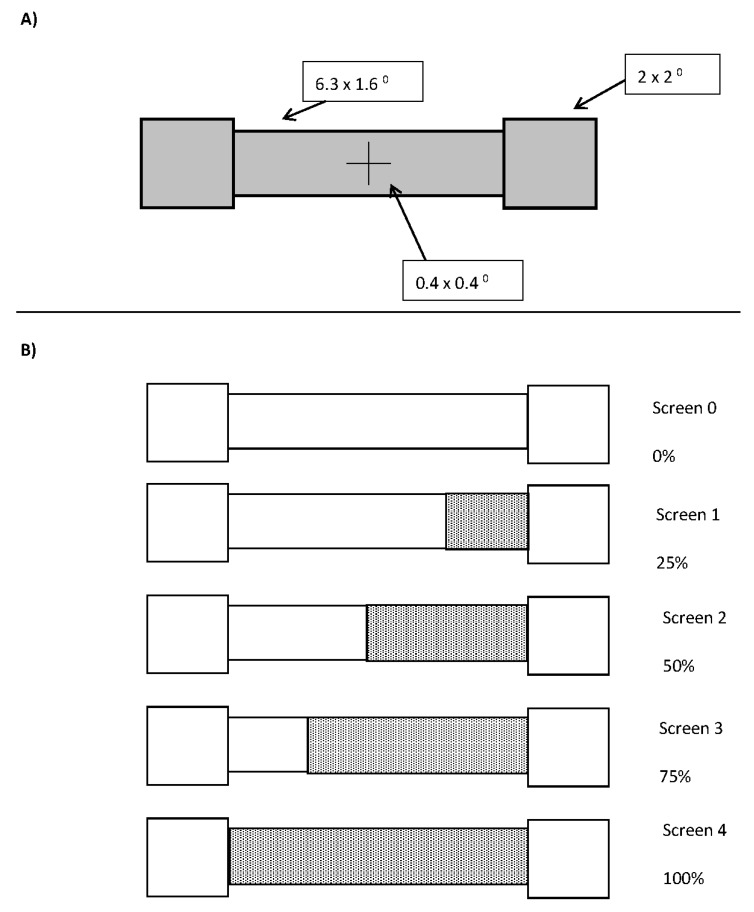
Schematic of (**A**) the stimulus display with measurements in degrees of visual angle and (**B**) the real leftward moving colour change at the slowest speed. The medium speed changed the bar in three segments, and the fast speed changed the bar in two segments. No motion changes the bar on a single screen refresh.

The barbell display comprised of solid grey squares that subtended 2 degrees of visual angle centred 4.15 degrees of visual angle to the left and right of a black fixation cross on a dark grey background. The bar between the two ends was 1.6 degrees of visual angle in height and 6.3 degrees of visual angle in width. A schematic of the display can be seen in Figure 1A.

#### 2.1.4. Procedure

A trial began with the presentation of a fixation cross and the barbell for 1000 milliseconds. In one-third of the trials, the left box changed to white (flash) for 50 milliseconds, and then returned to its original luminance. In one-third of the trials, the right box flashed for 50 ms. In the remaining third of the trials, neither box changed during this 50 ms interval. 

Upon flash offset, there was either a 16.7, 166.7, 316.7, or 466.7 ms delay (1 screen, plus 0, 150, 300, or 450 ms) at which point the bar changed colour to either red (for half the participants) or green (for the remaining half) over the course of 1, 2, 3, or 4 screen refreshes (all at once, in halves, thirds, or quarters, respectively). In half of the changes, when the colour changed over multiple screens, the direction was from left to right and in the other half from right to left, resulting in 84 different combinations of the seven levels of real motion (slow left, medium left, fast left, no motion, fast right, medium right, and slow right), three flash conditions (left, right, and none) and the four delays. There were 10 trials at each combination, resulting in 840 trials. A depiction of a slow left colour change is shown in Figure 1B.

Participants were instructed to indicate the direction in which the colour appeared to spread over the bar by pressing the 4 or 6 key on the number pad of the computer keyboard to indicate to the left or right, respectively. If they did not know in which direction the colour spread, then they were specifically asked to guess and to try and distribute their guesses between left and right equally rather than to choose a default response. Participants were reminded to make their choices quickly while maintaining accuracy in their choice of response. Finally, they were able to take a short self-timed break after completing every 210 trials.

### 2.2. Results

Trials in which a decision was made in less than 200 ms (0.15%) were considered anticipations and those over 2000 ms (0.45%) were considered reflective of distractions; both anticipations and distractions were discarded as were trials in which no response was made within 4000 ms (0.37%), resulting in data from over 99% of the trials being retained for analysis. 

#### 2.2.1. Percept Scores

**Figure 2:**
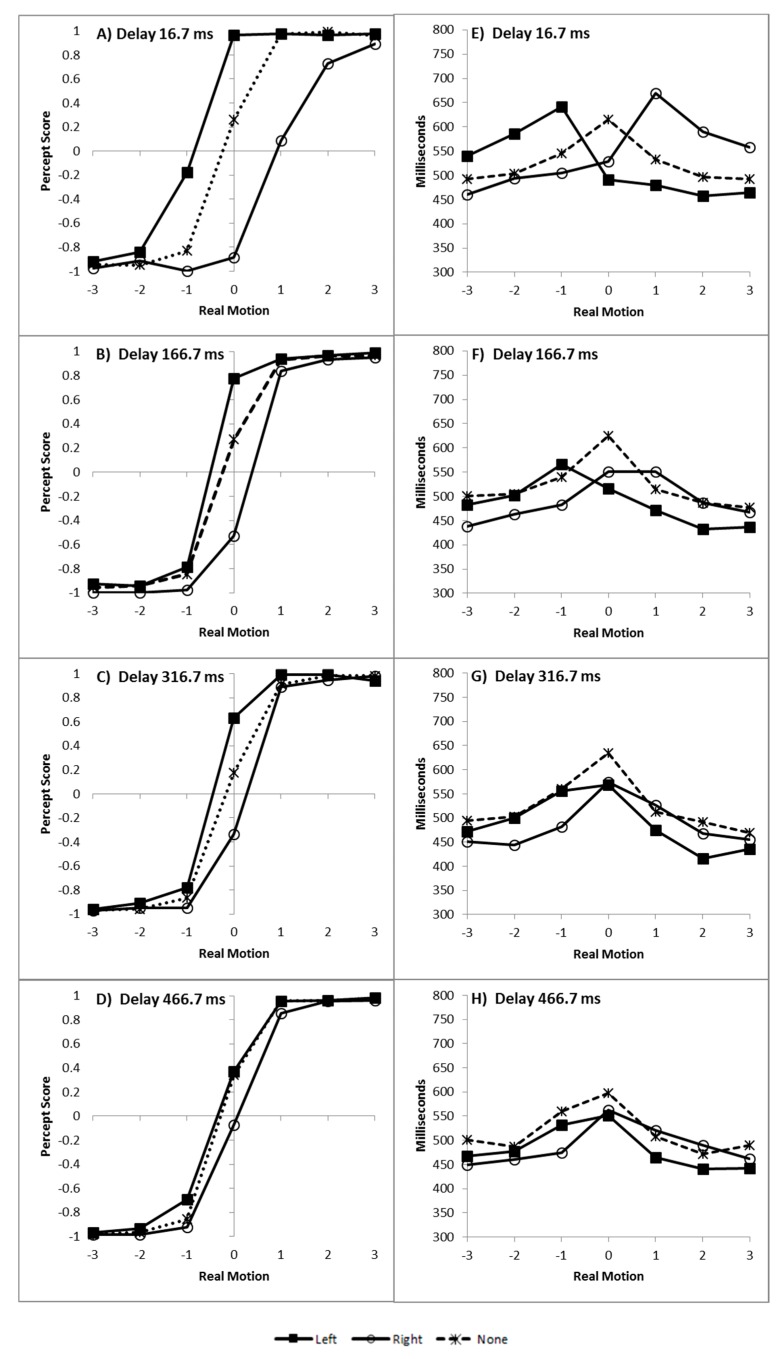
Mean perception scores (**A**–**D**) and decision times (**E**–**H**) from Experiment 1 for left (solid lines with filled squares), right (solid lines with open circles), and no flash conditions (dashed lines with asterisks) for cue-bar ISIs of 16.7 ms (A and E), 166.7 ms (B and F), 316.7 ms (C and G), and 466.7 ms (D and H).

Left responses were scored as −1 and right responses as +1 [6,7,8,10,11,12] and the mean score was plotted as a function of real motion (coded as −3 through to +3, for slow left through to slow right), flash location (left, right, or none) and delay, as can be seen in Figure 2A–D.

**Figure 3:**
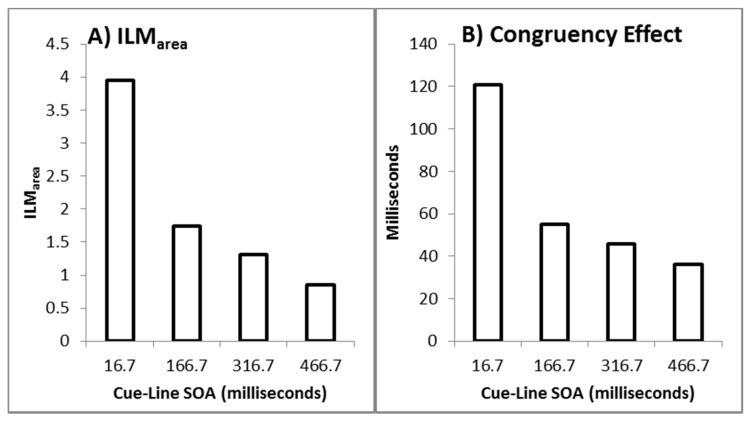
Mean (**A**) illusory line motion (ILM_area_) and (**B**) decision time congruency effect from Experiment 1 for cue-bar ISIs of 16.7 ms, 166.7 ms, 316.7 ms, and 466.7 ms.

As recommended by Han and Zhu et al. [11], _flash_ILM was quantified as the area between the left and right curves, allowing the illusion to be quantified as a single value (ILM_area_), shown in Figure 3A. ILM_area_ was then analysed in a within-subjects ANOVA with delay as a factor, which showed a main effect of delay (F_(3,69)_ = 78.121, *p* < 0.001, pH0|D < 0.01, MSE = 0.577, η_p_^2^ = 0.773; M = 3.946, 1.743, 1.314, 0.851, for delays 16.7, 166.7, 316.7, and 466.7 ms, respectively, with single sample t_(23)_ = 14.67, 10.53, 6.21, and 4.01, all *p* < 0.01 and pH0|D < 0.01 all very strong evidence against the null hypothesis, respectively). Planned contrast analysis was employed to look at trends over increasing delay intervals, resulting in linear (F_(1,23)_ = 127.622, *p* < 0.001, pH0|D < 0.01 very strong evidence against the null hypothesis, MSE = 0.887, η_p_^2^ = 0.847), quadratic (F_(1,23)_ = 33.711, *p* < 0.001, pH0|D < 0.01 very strong evidence against the null hypothesis, MSE = 0.539, η_p_^2^ = 0.594), and cubic (F_(1,23)_ = 12.835, *p* = 0.002, pH0|D = 0.02 strong evidence against the null hypothesis, MSE = 0.306, η_p_^2^ = 0.358) trends. If the mean ILM_area_ values are plotted as a function of the natural log of the delay, these show a strong linear function y = −0.9185x + 6.5183, r^2^ = 0.9976, pH0|D < 0.01, very strong evidence against the null hypothesis.

#### 2.2.2. Decision Time Congruency Effect

The time taken to decide in which direction the colour appeared to move is plotted as a function of real motion (coded as −3 through to +3, for slow left through to slow right), flash location (left, right, or none) and delay, and can be seen in Figure 2E–H.

The conditions in which the illusory motion signal would conflict with the real motion signal (left flash with leftward real motion and right flash with rightward real motion) are considered incongruent trials while those where the illusory motion and real motion signals would be expected to be in the same direction (left flash with rightward real motion and right flash with leftward real motion) are considered the compatible trials. The mean difference between the incompatible and compatible trials constitutes the decision time congruency effect [6,8,11,12] and can be seen in Figure 3B. The decision time congruency effect was analysed in a within-subjects ANOVA with delay as a factor. The decision time congruency effect differed between delays (F_(3,69)_ = 21.275, *p* < 0.001, pH0|D < 0.01 very strong evidence against the null hypothesis, MSE = 1656.120, η_p_^2^ = 0.481; M = 121, 55, 46, and 36 for blocks 1 through 4, respectively. All single sample *t*-tests t_(23)_ > 4.00, *p* < 0.001, pH0|D < 0.01, all very strong evidence against the null hypothesis). Planned trend analysis indicates a linear (F_(1,23)_ = 39.285, *p* < 0.001, pH0|D < 0.01 very strong evidence against the null hypothesis, MSE = 2112.266, η_p_^2^ = 0.631) and quadratic trend (F_(1,23)_ = 14.067, *p* = 0.001, pH0|D = 0.02 strong evidence against the null hypothesis, MSE = 1331.758, η_p_^2^ = 0.379), but no cubic trend (F_(1,23)_ = 2.616, *p* = 0.119, pH0|D = 0.57 weak evidence in favour of the null hypothesis, MSE = 1524.336, η_p_^2^ = 0.102). If the mean decision time congruency effect values are plotted as a function of the natural log of the delay, these show a strong linear function y = −25.588x + 191.24, r^2^ = 0.9910, pH0|D < 0.01, very strong evidence against the null hypothesis.

#### 2.2.3. Relationship between the Measures

**Figure 4:**
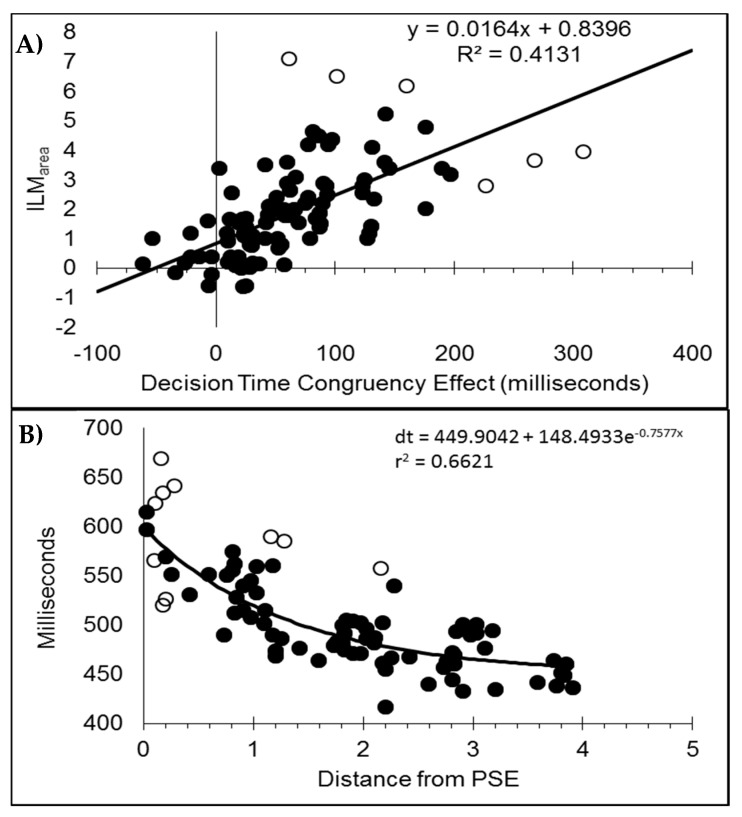
Scatterplots showing the (**A**) correlation between the decision time congruency effect and ILM_area_, and (**B**) the exponential distance decay function relating the decision times to the distance from the point of subjective equality from Experiment 1. The open symbols indicate data pairs that exceeded the Cook’s Distance inclusion criterion.

After dropping six data pairs with excessive influence as determined by Cook’s distance exceeding 0.0635 (4/n), [6,8,11,12], the decision time congruency effect was correlated with ILM_area_ (r_(88)_ = 0.6427, *p* < 0.001, pH0|D < 0.01, very strong evidence against the null hypothesis), with the scatterplot shown in Figure 4A. If all the data pairs are included, the correlation is r = 0.5955. Note, this relationship has shown a wide variety in the pattern of results for _flash_ILM, ranging from non-significance [6 Experiment 1,11], weakly related [12], or strongly related [6 Experiment 2,8] and the current finding.

#### 2.2.4. Decision Times as a Function from the Point of Subjective Equality

The decision times were plotted as a function of distance from the point of subjective equality and then fitted to a distance decay function after removing 10 data pairs with Cook’s distance larger than the inclusion criterion (4/84 = 0.0476) as shown in Figure 4B. The point of subjective equality (PSE) was determined by least squares fitting of the logistic regression equation y = 2[(e^−ax+b^)/(e^−ax+b^ + 1)] − 1, which is scaled to the range of the percept scores [6,8,11,12]. This resulted in a distance decay function of dt = 449.9042 + 148.4933e^−0.7577x^, r = 0.8137, pH0|D < 0.01, which is very strong evidence against the null hypothesis. Note, if all the data pairs are included, the distance decay function becomes dt = 446.1727 + 159.7055e^−0.6924x^, r = 0.8009.

### 2.3. Discussion

The spreading of the colour change over the bar was detected as illusory motion as indicated by both the inaccuracy of the null and the very strong evidence in favour of non-zero values for both ILM_area_ and decision time congruency effect at all SOAs. In addition, the illusory motion could be cancelled to various degrees by actually spreading the colour towards the flash. Decision times to indicate the direction of motion were longer as the percept scores approached zero, and were well described as an exponential distance decay function away from the point of subjective equality, replicating previous findings with onset and offset bars [6,8,11,12]. This increase in decision times for conditions approaching the point of subjective equality indicates that the direction of motion signal is being reduced and the information upon which the participants are making their left/right decision is approaching the response boundary [30]. Note, at the PSE on a trial by trial basis, there may be weak signals of randomly left or right motion reflecting typical variation, or there may be other non-directional forms of motion, such as PGM from both locations creating “inward motion”, or an outward expansion from the centre of mass (gamma motion), but neither of these motion signals lead to a consistent response choice and so are not under investigation. For clarity, the reduction of motion signals here should be taken to mean a reduction of the directional motion signals generated by the illusion and by the real motion present in the stimulus and not to mean the absolute absence of any sense of jitter or multidirectional motions.

The alternative interpretation of the PSE is that it reflects the point where the two alternative motion signals do not combine but are equally likely to win access to conscious perception, similar to the situation with ocular dominance where only one of two images reaches awareness. If this were the case, then participants would also choose each response equally often because the real and illusory motions are in opposite directions but they would be seeing strong directional motion on every trial equally split between the two directions. However, if this were the case, then the expectation would be for decision times at the PSE to be the average of the decision time for the real and illusory motion alone [7]. Since decision times at the PSE are slower than either, as when no motion is present (no motion, no flash trials), a winner-takes-all type situation is not supported by the data.

ILM_area_ decreased as the cue-bar SOA was extended, as predicted by a fading attentional gradient. In addition, the decision time congruency effect also diminished with increasing cue-bar SOA. While the percept scores and decision time congruency effect were correlated, as has been shown before [8], this is not always the case [6,11]. This relationship is thought to reflect how both the percept scores and the decision times will be influenced by the strength of the resulting motion signal rather than by the processes that result in the illusion itself [6]. Interestingly, _flash_ILM shows a rapid decrease between cue-bar SOAs of 66.7 and 216.7 ms, with a slower and steady rate of decline between SOAs of 216.7 and 516.7 ms. Whether this reflects an exponential decay of the attentional gradient or the interesting possibility of two processes contributing to ILM at the short interval, one of which fades rapidly leaving only the longer lasting process at the larger intervals, remains to be investigated. Two possible processes could be short lasting cue-bar sensory interactions between the cue and bar [31] and a longer lasting influence reflecting exogenous attention [32].

Regardless, Experiment 1 has established that ILM_area_ diminishes over increasing cue-bar SOAs, consistent with the predictions from the attentional gradient explanation for _flash_ILM. In addition, it has established that ILM_area_ detects the presence of _flash_ILM for a sufficient amount of time post-flash that the temporal separation required for the double-change condition of Experiment 2 is feasible.

## 3. Experiment 2

Experiment 1 demonstrated that ILM_area_ decreases over the time course in which one would expect the attentional gradient to be fading. This could simply reflect a coincidental correspondence in the time course of the fading of exogenous visual attention and the fading of some other process that is responsible for _flash_ILM. However, having established that _flash_ILM occurs over an extended time period, if we disrupt the attentional gradient by redistribution [20] and if _flash_ILM is not reduced, then this would be hard to reconcile with the attentional gradient theory. In contrast, if _flash_ILM is also reduced during the conditions when the asymmetry of the attentional gradient is reduced, then this would be consistent with the predictions derived from the attentional explanation for _flash_ILM. It should be noted that changing the colour of a fixation cross did not appear to draw attention away from a flashed location in a saccadic task but changing the colour of the bar to produce ILM did [22], so there is some evidence to argue against the idea that a colour change alone will draw attention away from the flashed location. Moreover, simply presenting a bar between two locations did not appear to result in a change in attentional asymmetry during a cuing task, consistent with the notion that it is the illusory motion that may redistribute attention over the display [20]. Whether attention shifts because of the colour change or in response to the illusory motion may be an interesting question but one that is tangential to the current investigation as the important point is that the gradient has been disrupted. 

### 3.1. Methods

#### 3.1.1. Participants

Twenty-four right handed male subjects between the ages of 18 and 32 (mean age 24.3 years) voluntarily gave informed written consent prior to participation in this study. All had normal or corrected-to-normal vision. The methods were approved by The University of Auckland Human Participants Ethics Committee.

#### 3.1.2. Apparatus

The same apparatus were used as in Experiment 1.

#### 3.1.3. Stimuli

The barbell display comprised of solid squares that subtended 2 degrees of visual angle centred 4 degrees of visual angle to the left and right of a black fixation cross on a dark grey background. The bar between the two ends was 2.0 degrees of visual angle in height and 6 degrees of visual angle in width. The boxes and barbell were a brighter shade of grey than the background.

#### 3.1.4. Procedure

The procedure was identical to that of Experiment 1 with the following changes. The longer two delay conditions were dropped. The 16.7 and 166.7 delay conditions were retained and are referred to as the early and late single change conditions as the bar would change to the target colour (red or green, equally divided over participants, with the other referred to as the distractor colour) to which they would make their response. 

A double change condition was then introduced to replace the removed trials from the two longer delay conditions. The double change condition was identical to the 166.7 ms single change condition except that immediately upon flash offset, the bar would change all at once to the distracter colour, which the participant was instructed to ignore. 

There were 10 repetitions of each of the 21 conditions (three flash conditions—left, right, none—by 7 levels of motion, slow left through to slow right, as per Experiment 1) for both single change conditions, and 20 repetitions of each of the 21 conditions for the double change condition, for a total of 840 trials. The trials were presented in a random order, with all the conditions intermixed. 

Of the 20,160 trials from all participants, 61 (0.30%) with response times less than 200 ms and 345 (1.71%) with response times over 2000 ms were discarded as anticipations or distractions, respectively. Six trials (<0.01%) were discarded as having terminated with no response or by pressing an invalid key, leaving 97.96% of the trials available for analysis. The percept scores and mean decision times were calculated as in Experiment 1. 

### 3.2. Results

**Figure 5:**
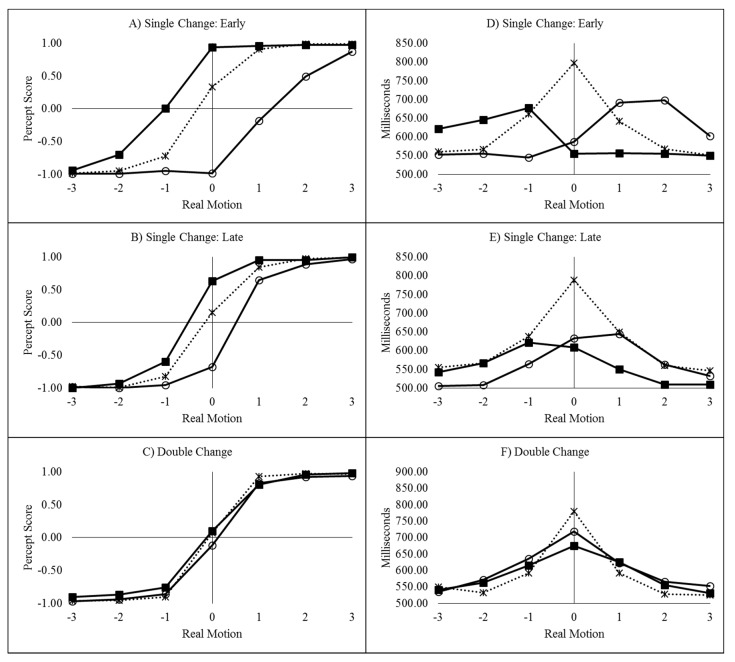
Mean perception scores (**A**–**C**) and decision times (**D**–**F**) from Experiment 2 for left (solid lines with filled squares), right (solid lines with open circles), and no flash conditions (dashed lines with asterisks) for the early (A and D), late (B and E), and double (C and F) change conditions.

The mean percept scores as a function of real motion and flash location for the single-early, single-late, and double change conditions can be seen in Figure 5A–C. 

#### 3.2.1. ILM_area_

**Figure 6:**
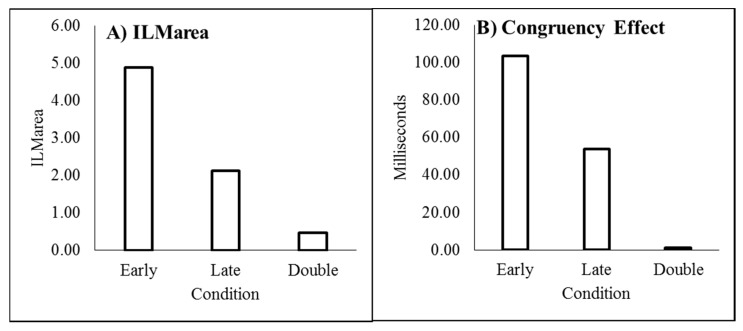
Mean (**A**) ILM_area_ and (**B**) decision time congruency effect from Experiment 2 for the early, late, and double change conditions.

ILM_area_ was analysed in a within-subjects analysis of variance (ANOVA) to determine whether the conditions (early, late, and double) differed. The mean ILM_area_ for each condition can be seen in Figure 6A. This indicated that ILM_area_ differed between conditions (F_(2,46)_ = 83.060, *p* < 0.01, pH0|D < 0.01 very strong evidence against the null hypothesis, MSE = 1.430, η_p_^2^ = 0.783; M 4.872, 2.118, and 0.468 for early, late, and double, respectively). Each condition was then tested in a single sample *t*-test to determine whether the ILM_area_ differed from zero, which it did for the single-early (t_(23)_ = 11.132, *p* < 0.001, pH0|D < 0.01 very strong evidence against the null hypothesis) and single-late (t_(23)_ = 7.524, *p* < 0.001, pH0|D < 0.01 very strong evidence against the null hypothesis) conditions but not the double change (t_(23)_ = 1.534, *p* = 0.139, pH0|D = 0.60, weak evidence in favour of the null hypothesis) condition [33].

#### 3.2.2. Decision Time Congruency Effect

The mean decision times as a function of real motion and flash location for the single-early, single-late, and double change conditions can be seen in Figure 5D–F. The difference between conditions where real motion and ILM would conflict (leftward real motion combined with a left flash and rightward real motion combined with a right flash) and conditions where real and illusory motion would be expected to be in the same direction (leftward real motion combined with a right flash and rightward real motion combined with a left flash) comprises the decision time congruency effect.

The decision time congruency effect, shown in Figure 6B, was analysed in a within-subjects analysis of variance (ANOVA) to determine whether the conditions (early, late, and double) differed. This indicated that the decision time congruency effect differed between conditions (F_(2,46)_ = 22.396, *p* < 0.01, pH0|D < 0.01 very strong evidence against the null hypothesis, MSE = 2795.804, η_p_^2^ = 0.493; M 103, 54, and 1 ms for early, late, and double, respectively). Each condition was then tested in a single sample t-test to determine whether the decision time congruency effect differed from zero, which it did for the single-early (t_(23)_ = 8.886, *p* < 0.001, pH0|D < 0.01 very strong evidence against the null hypothesis) and single-late (t_(23)_ = 4.731, *p* < 0.001, pH0|D < 0.01 very strong evidence against the null hypothesis) conditions but not the double change (t_(23)_ = 0.084, *p* = 0.934, pH0|D = 0.83 positive evidence in favour of the null hypothesis) condition.

#### 3.2.3. Relationship between ILM_area_ and Decision Time Congruency Effect

**Figure 7:**
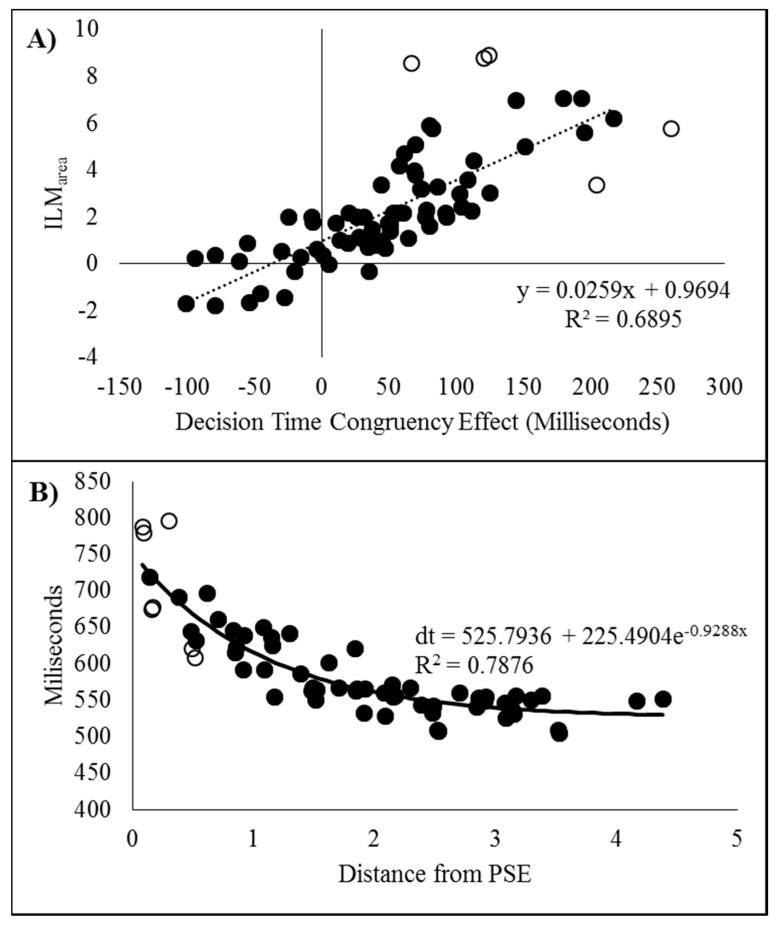
Scatterplots showing the (**A**) correlation between the decision time congruency effect and ILM_area_ and (**B**) the exponential distance decay function relating the decision times to the distance from the point of subjective equality from Experiment 2. The open symbols indicate data pairs that exceeded the Cook’s Distance inclusion criterion.

To further examine the possible relationship between the decision time congruency effect and ILM_area_, the two values were correlated. As recommended by Han and Zhu et al. [11], the correlation is reported after discarding the five data pairs found to have an excessive influence on the correlation based upon having a Cook’s distance larger than the cut-off criterion of 0.056; 4/n (n = 72). The two measures were found to be related (r_(65)_ = 0.8304, *p* < 0.001, pH0|D < 0.01, very strong evidence against the null hypothesis) and the scatterplot can be seen in Figure 7A. If all the data points are included, then r = 0.7700.

#### 3.2.4. Decision Times as a Function from the Point of Subjective Equality

As with Experiment 1, the decision times were plotted as a function of distance from the point of subjective equality and then fitted to a distance decay function after removing seven data pairs with Cook’s distance larger than the criterion (0.063; 4/n, n = 63). The scatter plot can be seen in Figure 7B fitted to the decay function dt = 525.7936 + 225.4904e^−0.9288x^, r = 0.8875, *p* < 0.001, pH0|D < 0.01, which is very strong evidence against the null hypothesis. Note, if all the data pairs are included, the distance decay function becomes dt = 530.8908 + 232.7683e^−1.0532x^, r = 0.9004.

### 3.3. Comparison between Experiments 1 and 2

Both experiments 1 and 2 presented single colour changes at short and long cue-bar ISIs, namely 16.7 and 166.7 ms. ILM_area_ and the decision time congruity effects were measured at these intervals in both experiments. The data was compared between experiments to determine whether the quantities were replicated despite the other changes in the experimental paradigms. 

The mean percept scores were analysed in a mixed-factor 2-way ANOVA with ISI (short and long) as a within-subjects factor and the experiment as a between groups factor. There was a main effect of ISI (F_(1,46)_ = 141.758, *p* < 0.001, pH0|D < 0.01 very strong evidence against the null hypothesis, MSE = 1.040, η_p_^2^ = 0.755; M = 4.409 and 1.931 for short and long ISI, respectively) which did not interact with the experiment (F_(1,46)_ = 1.748, *p* = 0.193, pH0|D = 0.67 weak evidence in favour of the null hypothesis, MSE = 1.040, η_p_^2^ = 0.037; M = 3.946 and 1.743 for Experiment 1 at short and long, respectively and 4.872 and 2.118 for Experiment 2’s single change conditions at the short and long ISI, respectively). Finally, there was no main effect of the experiment (F_(1,46)_ = 2.982, *p* = 0.091, pH0|D = 0.60 weak evidence in favour of the null hypothesis, MSE = 3.407, η_p_^2^ = 0.061; M = 2.844 and 3.495 for Experiment 1 and 2, respectively).

The mean congruency effects were similarly analysed in a mixed-factor 2-way ANOVA with ISI (short and long) as a within-subjects factor and the experiment as a between groups factor. There was a main effect of ISI (F_(1,46)_ = 39.410, *p* < 0.001, pH0|D < 0.01 very strong evidence against the null hypothesis, MSE = 2020.262, η_p_^2^ = 0.461; M = 112.008 and 54.410 for short and long ISI, respectively) which did not interact with the experiment (F_(1,46)_ = 0.792, *p* = 0.378, pH0|D = 0.82 positive evidence in favour of the null hypothesis, MSE = 2020.262, η_p_^2^ = 0.017; M = 120.657 and 54.893 for Experiment 1 at the short and long ISI, respectively and 103.358 and 53.927 for Experiment 2’s single change conditions at the short and long ISI, respectively). Finally, there was no main effect of the experiment (F_(1,46)_ = 0.405, *p* = 0.528, pH0|D = 0.85 positive evidence in favour of the null hypothesis, MSE = 4947.045, η_p_^2^ = 0.009; M = 87.775 and 78.643 for Experiment 1 and 2, respectively).

In short, there was no evidence to suggest that there was any substantial influence of the changes in protocol with regards to either measure. 

## 4. Discussion

The results from the current experiments replicate the finding that _flash_ILM can cancel the perception of real motion [6,7,8,11]. The size of the illusion decreases as the cue-bar ISI increases as shown by the reduction in ILM_area_ with increasing cue-bar ISI. However, as also shown in Experiment 1, the evidence for the illusion continues to be very strong with the null making inaccurate predictions at the late interval when there was only a single colour change. To the extent that attention is responsible for the illusion, this finding is consistent with the fact that the attentional costs plus benefits of non-informative cues reduce over time [34], reflecting a temporal decay of the attentional gradient. 

Critically, during the double change condition of Experiment 2, the null prediction was not inaccurate and the evidence was weakly (ILM_area_) or positively (decision time congruity effect) in favour of the null at the late interval, indicating _flash_ILM has been reduced to non-detectable levels. In the double change condition, the distractor colour change occurred at an interval when _flash_ILM occurs, as in the early single change condition. This distractor colour change would produce _flash_ILM that disrupts the attentional gradient, either because attention might redistribute over the display following the illusory motion [20] or attention may simply shift to the centre of the bar due to the change in colour [35]. However, colour changes at fixation do not seem to result in an attentional shift in other _flash_ILM paradigms [22]. Regardless, the critical aspect is that the colour change should redistribute attention such that the second colour change occurs along a much less asymmetrically distributed attentional gradient.

Similar to Experiment 1, the decision time congruency effect also diminished with increasing cue-bar ISI. The congruency effect was also correlated with the size of the illusion. This relationship has been unstable, however, and is not always found [11]. This correlation is thought to reflect the fact that both ILM_area_ and the congruency effect are influenced by the strength of the motion signal, but while ILM_area_ reflects processes up to the generation of the motion signal, the congruency effect reflects the subsequent influences on later decision processes [6,12]. 

In addition, the decision times decreased as an exponential distance decay function based upon the distance from the point of subjective equality (PSE). This was also found in Experiment 1 and the same pattern is reported elsewhere [6,10,11,12]. This finding indicates that the motion signal that results from _flash_ILM and real motion reduces when the two motions are in opposite directions because this moves the resulting motion signal closer to the response boundary [30] and does not correspond to the PSE reflecting equality in a perceptual winner-take-all type competition between the illusory and real motion (see [7,10] for further discussion on this). Moreover, if a third “no motion” response option is included, this choice is also maximal at the PSE [11], which is not consistent with participants perceiving either the real motion signal or the illusory motion signal. Therefore, the speed of the decision appears to reflect the strength of the combined motion signal, which results in a tendency for one to cancel the other when in opposite directions, resulting in a net reduction in the directional motion.

The data from Experiment 1, with ILM_area_ decreasing as a function of the cue-bar ISI, is also consistent with the attentional gradient explanation given that the costs plus benefits of exogenous attention reduce over time [36]. This corresponds nicely with the finding that an individual’s _flash_ILM_area_ is correlated to their costs plus benefits from an exogenous attention cuing task [8].

The findings from Experiment 2 showed that _flash_ILM fell below detectable levels when the target colour change was preceded by a distracter colour change. This finding is also consistent with predictions derived from the attentional cuing literature. Hamm and Klein [20] investigated the costs plus benefits during a cuing task and concluded that attention automatically followed the illusory motion by spreading over the display to encompass the entire line. According to Hamm and Klein [20]’s spreading of attention theory, following the presentation of the distractor colour, exogenous attention should no longer be as strongly asymmetrically distributed towards one end of the bar as the _flash_ILM generated by the distractor colour would redistribute attention more evenly. Therefore, at the late interval, when the target colour change occurs, the colour change occurs along a much reduced gradient of attention. Importantly, the non-detection of _flash_ILM in the double change condition cannot be attributed simply to the passing of time as _flash_ILM was detected at this time in the single change condition. In addition, Experiment 1 demonstrated that _flash_ILM remains detectable even well after the late interval of Experiment 2. Therefore, during the double change condition, it is suggested that the initial distractor change in colour either drew attention to the centre of the bar, or the illusory motion stretched attention over the bar [20], and so the asymmetrical distribution of attention to the flash location was disrupted.

In summary, these findings replicate the finding that _flash_ILM can be produced in a display where the bar is initially presented and takes the form of a directional painting illusion [12,22]. The finding that _flash_ILM reduces with increasing temporal separation between the cue and the bar is consistent with the decay of exogenous attention over time [36]. The results are also consistent with the suggestions of Hamm and Klein [20], who suggested that attention tracks _flash_ILM and stretches over the entire display. At what point in time attention begins to shift and whether it is shifting due to the illusory motion or simply due to the colour change in the bar remains open for further investigation. 

This brings us to a consideration of the role of consciousness. The following discussion reflects some thoughts and ideas that have arisen in part from the program of investigation of illusory line motion of which the current studies are a part. It is, however, intended and recognised as moving beyond the normal bounds of what a specific study is designed to specifically investigate and so the connections between the ideas and the current data are presented as exploratory food for thought. The following section will, therefore, be presented in the format of a discussion, describing the hypotheses and the resulting data analysis that was performed to determine whether the resulting predictions can be viewed as plausible, if not unique. This is, in part, to avoid implying that the following examination was a priori intended during the designing of these experiments. Therefore, the analyses that follow should be read as observational and the discussion of them as hypotheses, and not as evidence and conclusions.

With regards to consciousness, one suggestion is that it is epiphenomenal and nothing more than emergent qualia that have no consequence on the underlying machinery involved in decision making; the decision is already made, and the quale of self-volition arises much later [37] but is an inaccurate representation of a system that simply is on its way to responding. Some have argued that it could be entirely possible for a non-conscious experiencing “zombie” to produce indistinguishable behavioural outputs [38]. These notions effectively describe consciousness as a ghost in the machine, where the qualia of conscious experience, including that of self-volition, arise from the underlying neural system but the qualia do not reflect a system that actually self-governs its action. To use a slightly different metaphor from that of zombies, let us imagine we have a wire stretched between two points upon which we hang a small weight. Our measurement is the distance from the floor to the weight. As we apply heat, the wire will become increasingly malleable and the weight will move closer to the floor. As more physical energy is applied to the wire, there will reach a point where the wire glows. However, because glowing is a property that exists in the observer and has no impact upon the physical aspects or behaviour of the wire, the change in the distance of our weight to the floor will continue to follow a predictable path of expected measurements derived from the pre-glow point observations. This glow point is intended as a metaphor for the point where neuronal activity driven by the stimulus results only in epiphenomenal conscious awareness; consciousness happens but there are no consequences for the system from which it emerges. So, crossing the threshold from non-aware to aware has no impact upon our objective measurements of the physical system, just like glowing does not change how the wire responds to increases in applied heat.

An alternative is that consciousness is a fundamental change of state that has direct consequences upon the physical system. Continuing with the wire metaphor, this would be the point where the wire reaches a temperature where it changes in state from solid to liquid, the melting point, with obvious consequences with respect to the weight’s distance from the floor [39]. Here, consciousness is suggested to entail some fundamental change in the state of the underlying neuronal system that responds to stimulus input, and therefore allows for new consequences to emerge. With regards to the current discussion, a change of state could consist of the synchronization of neuronal activity [40,41], a neuronal system entering into sustained activity [42], spreading of activity to create a larger network [43], activity within specialised neuronal cells (the newly discovered rosehip neurons; [44]), some as yet undiscovered change in how individual neurons transmit information, or even perhaps something beyond our current understanding or capabilities to measure such as an interaction with proposed “dark matter”. No matter how such a change of state manifests physically, the important point is that there is some physical change of state in the underlying neural mechanism. Therefore, the system works differently when aware compared to unaware, and this allows for the possibility that the neuronal system can self-generate or self-modify responses, which is a property that would manifest in the quale of volitional intent. In a change of state model, the quale of volitional intent would simply be an accurate representation of a system where self-modification by the change in state has occurred. In short, if consciousness is best described metaphorically as a melting point, then this is to say that the mechanism that produces consciousness is able to influence the physical system differently than when non-conscious, and therefore describes a system that partakes in the process of decision making. While this does not answer Chalmer’s “hard problem” of why qualia arise [45], it seems likely that the first step to answering that question requires understanding what systems are involved, and whether a change from non-aware to aware is more akin to crossing a “glow point” or “melting point” type of threshold seems fundamental to that understanding. 

While it has been shown that brain activity can be qualitatively different between stimuli of which the observer is aware and not aware [43,46], the current investigation is focused on whether or not predictions derived from a change in state hypothesis are observed in behavioural measures. It is not intended as a full comparison between the “glow point” and “melting point” possibilities. Due to the exploratory nature of this question, some initial starting premises have been adopted and from which the predictions are then derived. The first premise of a change in state model is that both state 1 and state 2 are capable of resulting in a response that is better than chance. It need not be that both states are equally accurate, only that both states are capable of producing performance better than chance.

Second, the system under investigation cannot be controlled to the same extent as the wire in our metaphor. It is as if the properties of our “wire”, so to speak, are constantly changing, such that the melting point is not a constant from moment to moment. In short, the second premise is that the threshold will vary moment by moment. 

Third, any given response or decision is the result of either output from state 1 or state 2. If the threshold is not exceeded, then the response generated reflects the operation of state 1. However, if the threshold is exceeded, then state 2 is activated and the response reflects state 2. It is important to keep in mind the second premise that states the threshold can vary moment to moment.

Whether or not the threshold is exceeded will be related to the strength of our stimulus signal. This is partially under our control in so much that we can change the physical intensity of the stimulus—in this case we can change the speed of the actual motion. However, the important signal strength for our consideration is not the physical stimulus, but rather the strength this stimulus creates within an individual, which can vary between participants. This last claim is hardly controversial as it is simply pointing out that different people will have different degrees of sensitivity to the same physical stimulus input as evidenced by the fact that such measures of signal intensity like d’ are not constants but show population variation. Equating the psychological stimulus strength would involve finding the stimulus intensity for each participant that results in a constant d’. This is counter to the typical experimental set up where it is the physical stimulus intensity that is controlled and the psychological signal is allowed to vary between participants. An example that highlights the importance of equating the psychological strength of the signal in contrast to the objective physical strength of the signal is how proposed tonal memory impairments associated with congenital amusia vanish when perceptual difficulty is equated on a participant by participant basis [47]. The fourth premise, therefore, is that as the psychologically relevant signal strength increases the probability that it exceeds the threshold and the response is generated by state 2 will increase. Note, because the psychological signal strength is presumed to be related to the physical input, equating stimulus conditions on the former still means the psychological signal will increase when stimulus intensity increases, despite the fact that different participants will convert different stimulus intensities to different psychological stimulus strengths. As a minimum, therefore, equating participants to a common zero value of psychological signal strength (signal strength hereafter) is important.

Finally, the fifth premise is that both state 1 and state 2, being capable of generating responses, will do so more quickly as the signal strength increases. However, even though the mean decision times will get systematically faster, the variability of the responses will generally be constant and the distribution of decision times around the shifting mean will not change as the state becomes more strongly active by increasing stimulus strengths. 

This last premise, with regards to the distribution shapes, is the only truly controversial starting point. Without this constraint, however, there are too many free parameters available for the subsequent analysis. The thinking behind this premise is that the variation in trial by trial response times is primarily due to the moment by moment variation of brain activity into which the stimulus signal is introduced. Particularly fast decisions may reflect the chance occurrence of a stimulus being presented when all systems through which stimulus processing proceeds happen to align in a beneficial way, and particularly slow decision times may reflect the occurrence of when more systems are generally in a state that prolongs processing. As a change of state could impact a wide range of brain activity, it is plausible to suggest that the different states will produce different distributions of response times.

Therefore, to the extent that the following can be considered an actual test of a hypotheses, then the hypothesis under investigation could be phrased as “If we presume the distribution of response times for state 1 and state 2 are constant despite changes in the means, can a two state model explain the decision times and the percept scores from the current illusory line motion tasks?” We will effectively start with this premise and examine how much of the data can be explained as a result.

To ease the following descriptions, the phrase “experimental conditions” will be used to refer to the flash (left, right, none) and delay (E1: the four cue-bar ISIs; E2: the early, late, and double conditions) aspects of the design after collapsing over the real motion aspect of the designs. Experiment 1, therefore, has a 3 × 4 matrix and Experiment 2 has a 3 × 3 matrix of experimental conditions.

The first step of the analysis is to convert our physical measures of motion into psychological signal strengths. This was achieved by finding an individual’s point of subjective equality over motion for each experimental condition. For example, a participant’s PSE for a left flash at delay 1 in Experiment 1. The signal strength for each level of real motion was calculated as the absolute distance of the real motion value from this point of subjective equality. So, if the PSE = −0.8, then motion condition 0 (no motion) would have a signal strength of 0.8 and motion condition −1 (fast left) would have a signal strength of 0.2. This allowed for the recoding of every trial, regardless of experimental condition, in terms of an individual participant’s average signal strength. For all participants, this resulted in a range of signal strengths from 0 to 6, inclusively.

In addition, because the signal strength ignores direction of motion, responses were recoded as +1 if the response corresponded to the signal’s direction and −1 if they did not. Meaning, if the real motion fell to the left of the PSE, a left response was considered in correspondence with the signal and coded as +1, while a right response would be considered non-corresponding and coded as −1. 

The second step was to attempt to minimise response time variation between participants and experimental conditions. This was done by subtracting each participant’s mean decision time for a given experimental condition from all of the motion trials within that condition. This was to minimise the influence of such things as warning effects as the time between a flash and bar onset increased and individual differences in overall response speed.

The range of signal strengths, 0 to 6, was then divided in 100 equally spaced bins, with the first bin containing all trials with a signal strength less than or equal to 0.06, the second being above 0.06 but less than or equal to 0.12, and so forth, with the last bin containing the trials above 5.94 and less than or equal to 6. The 39,706 trials retained for analysis in Experiment 1 and 2 were then distributed in these bins and the mean signal-based percept scores and decision times were calculated for each bin. Upon examination, it was found that the 1898 trials (4.8% of the total) in the 41 bins above 3.54 were sparsely distributed, with nine of the bins containing no trials at all. The lower 59 bins contained the remaining 37,808 trials and had a minimum of 139 trials in each bin. The last 41 bins were collapsed into three bins with 723, 636, and 539 trials in each bin, with mean signal strengths of 3.75, 3.99, and 4.70, respectively. 

**Figure 8:**
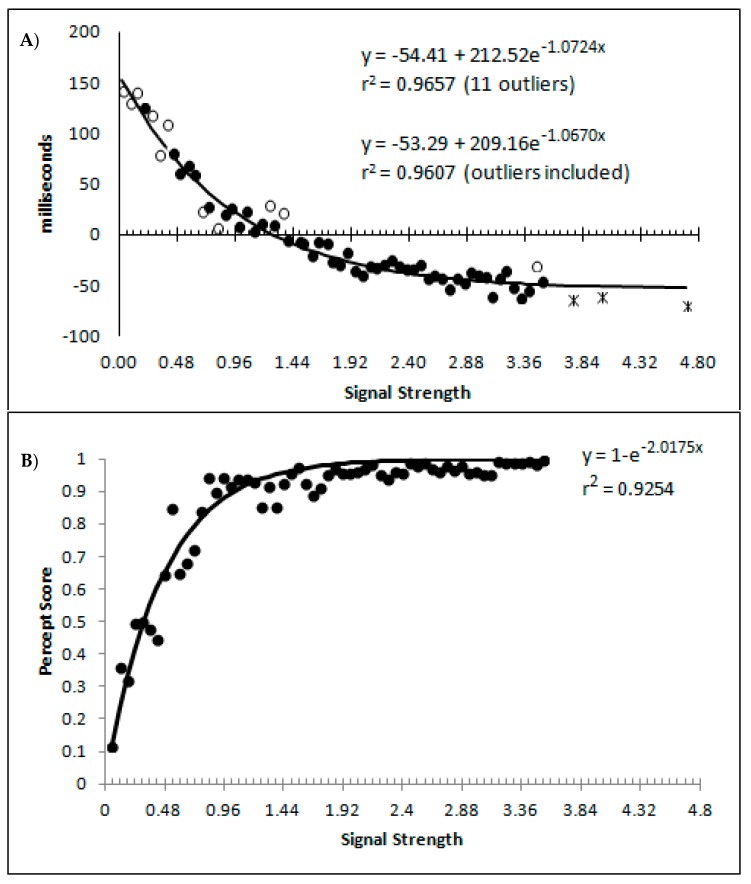
Shown as a function of signal strengths in bins of 0.06 width, are the (**A**) percept scores and (**B**) decision times for the combined data from Experiment 1 and 2. The decision times for each participant have had the main effects of cue location and delay condition removed.

The percept scores are plotted in Figure 8A below and they show a steady increase in the proportion of responses that were made in the direction that corresponds to the signal strength, with near perfect performance being reached around a value of 1. The corresponding mean decision times are shown in Figure 8B and continue to reflect a distance decay function. While the mean decision times for the collapsed data from the sparsely sampled upper 41 bins are shown for completeness, these were not included in the analysis. The decision times are deviations from individual participant means for conditions and blocks, so positive values are those slower than the mean, and negative values are faster than the mean. This is taken as an indication that the above transformations have not grossly distorted the data pattern from that which has been presented based upon group means and signal strengths based upon the group mean average data. 

At first blush, the decision time function is a smooth and apparently uninterrupted function of signal strength, which could be indicative of a single state with a “glow point” type model rather than a two states model. The smooth progression of both measures could be taken to reflect influences of increasing signal strengths with no evidence indicating any change in performance as would be expected if there were a change in state at some point between the weakest and strongest signals. With awareness likely to be absent for the weakest signals but present for the strongest, this pattern could be indicative of a “glow point” type of threshold with regards to awareness. 

However, smoothly curved response time functions can arise due to systematic changes in the contributions from two linear functions, as with the curved response time functions found during rotated letter mirror/normal discriminations [48,49,50,51]. Similarly, the two-state model involves predicting a steady change in the proportion of trials that are responded to while in state 1 or state 2 as a function of signal strength. It may be this changing of state contributions that provides an explanation for the shape of the data functions and why there is no immediate evidence of a sudden change in performance measures. This is the focus of the following discussion and it is a proof of concept exploration as to whether or not a change in state model can account for the observed data pattern.

**Figure 9:**
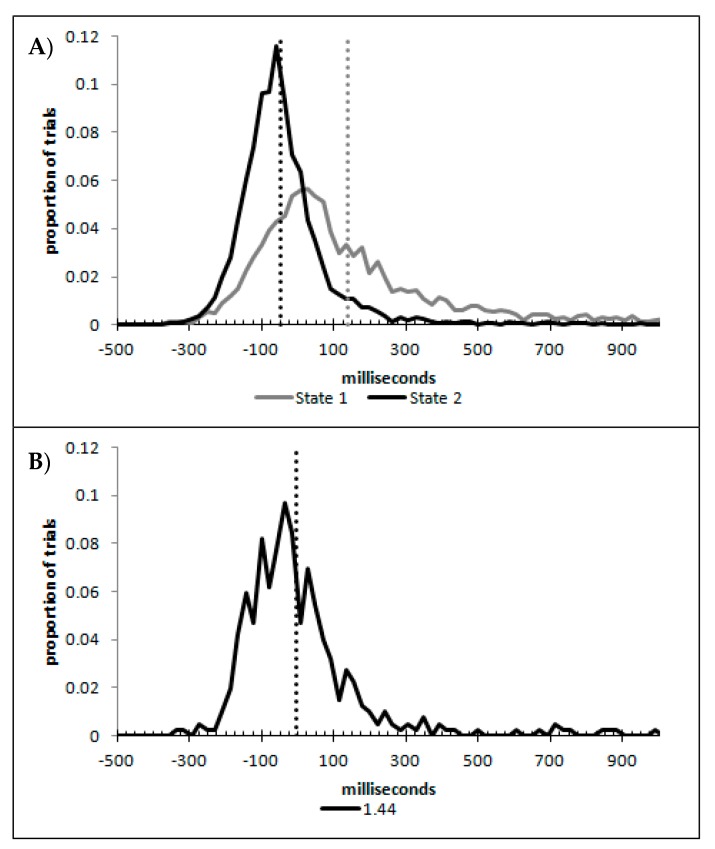
Density functions of (**A**) proposed state 1 (grey line) and state 2 (dark line) and (**B**) an example of a signal strength that may be a mixture of state 1 and state 2 responses.

In order to examine whether a mixture of two response time distributions, one reflecting state 1 responses and the other reflecting state 2 responses, can produce the observed distance decay function in decision times, we first require an estimation of those distributions. The mean decision times for the first three bins appeared relatively stable (141.02, 130.39, and 140.62 ms, respectively) and in total comprised 2866 trials. The decision time for each trial was adjusted to align each bin on the mean of the trials in all three bins (the bin mean was subtracted from each trial and the overall mean, 137.7 ms, added back in). These were taken as an estimate of state 1 responses based upon the signal strength being extremely small (≤0.18 from the PSE). Note, if a signal strength of 1 is taken to correspond to the difference in time between the presentation of the two halves of the bar at the fast motion speed, so 16.67 ms, this would correspond to a temporal onset asymmetry of 3.00 ms or less. To obtain a similar number of response times (2832), the last seven bins (not counting the 41 upper bins) were likewise combined to provide an estimate of the decision time distribution for state 2 responses with a mean of −46.53 ms. This includes all trials above the 3.12 signal strength cut off, and below or equal to 3.54 (again, in terms of the physical signal this would correspond to temporal onset asymmetries between 52 and 60 ms). The individual trials ranged between −572.67 and 1431.57 ms and this range was divided into 100 bins of 21.42 ms to create the density functions shown below in Figure 9a with vertical dashed lines indicating the means of each.

The next step was to create similar distributions for each of the individual bins; an example is shown in Figure 9B for the trials with a signal strength above 1.38 and below or equal to the upper cutoff of 1.44. This distribution was then centred on its mean: the mean for the bin was subtracted from all trials. The distributions for the state 1 and state 2 distributions were adjusted to their relative placement to the test bin’s mean (0), rounded to the nearest millisecond. These were then further shifted to move both the state 1 and state 2 distributions an additional 20 milliseconds further from 0. Effectively 20 milliseconds were added to each trial in the state 1 distribution, and 20 milliseconds were subtracted from each trial in the state 2 distribution. The density functions, using the response time bin cutoffs as previously described, were then recalculated for the distributions with the following final adjustment. Using the test bin distribution, the left most bins were combined to the point that at least 1% of the trials fell below a given cutoff point. For example, in a distribution of 500 decision times, if the fastest bin contained one response time, the next fastest contained none, the next contained two, and the fourth contained three, the cutoff for the fourth bin was used as the starting point, and would be considered to contain 6/500 trials, or 1.2% as the third bin would only cut off 3/500, or 0.6%. The density function was then calculated as usual until less than or equal to 1% of the total distribution remained, and all subsequent responses were placed in this bin. This decision was based upon the assumption that the extreme tails of the distributions are likely to be poorly represented combined with the desire to retain as much information about the distribution as possible. The density functions for the state 1 and state 2 distributions were then calculated using only the bins corresponding to the test distribution.

Next, the sum of the squared error between the test density function and the state 1 and state 2 distributions were then calculated for all bins in which the test distribution actually had observed data. Empty bins were presumed to reflect under-sampling of the true distribution. On average, there were 41.6 bins containing data, and 7.7 empty bins; these do not sum to a whole number because the number of bins changes as the test distributions change shape. The test distribution was left unchanged from this point forward. However, the means of the state 1 and state 2 distributions were then decreased and increased by 1 ms, respectively, and their density functions recalculated, and the process repeated until the mean of the state 1 distribution reached the starting point of the state 2 distribution and vice versa. Effectively, this is performing a brute force cross correlation between the test distribution and the state 1 and state 2 distributions independently. The mean that produced the lowest sum of squared error between the test distribution and the state 1 and state 2 distributions, and the corresponding error terms, were stored.

Finally, all combinations of the mean shifted state 1 and state 2 distributions were then combined by a weighted summation where the weightings for state 1 varied between x = 0% and x = 100% in 1% steps, and the weighting for state 2 was set to be 1 − x%. Meaning, in a 10% state 1 combination with 90% state 2, if the state 1 distribution predicted 6% of the response times should be in a particular bin while the state 2 distribution predicted that 10% should be in that same bin, these were combined as 0.1 × 0.06 + 0.9 × 0.10 = 0.096, or 9.6%. The sum of the squared errors was calculated for this weighted blended distribution and the combination of state 1 mean time, state 2 mean time, and the weighting between were retained for the combination with the lowest error term. The mean time for state 1 and 2 for the blended distribution do not necessarily correspond to the mean time for state 1 and 2 when tested singularly. This is likened to multiple regression, except based upon cross correlations and incorporates the second part of premise 5 into the analysis, that the distribution shapes do not change. Note, the initial single state cross correlations would be the situation where the weights are 1.0 to the state being tested and 0 to the excluded state. 

For each of the 59 signal strength bins, we now had three best fitting distributions, a single state 1, single state 2, and the blended distribution. Each distribution’s sum of squared errors was converted to r^2^ by dividing by the observed squared error by the total error of each bin’s distribution around the mean expected percentage over bins with data and subtracting this ratio from 1. Each r^2^ value was converted to a Bayesian information criterion value by the formula BIC = n ln(1 − r^2^) + k ln(n), where n was the number of bins containing data and k is the number of parameters. For the single distributions alone, a response time distribution can be modelled from the mean, standard deviation, and skew, and although we are using the observed values, each distribution was counted as three parameters, and an additional parameter is included to account for the residual error. For the blended distribution, which involves both state 1 and state 2 distributions, a percentage value for state 1 (the percentage value for state 2 is redundant with this), and residual error, makes k = 8. The bin was classified based upon the model producing the lowest BIC value (single state 1, single state 2, or dual). The presence of blended distributions, where either state 1 or state 2 responding could occur for the same signal strength, reflects premise 2, i.e., the threshold for a change of state varies due to background noise.

**Figure 10:**
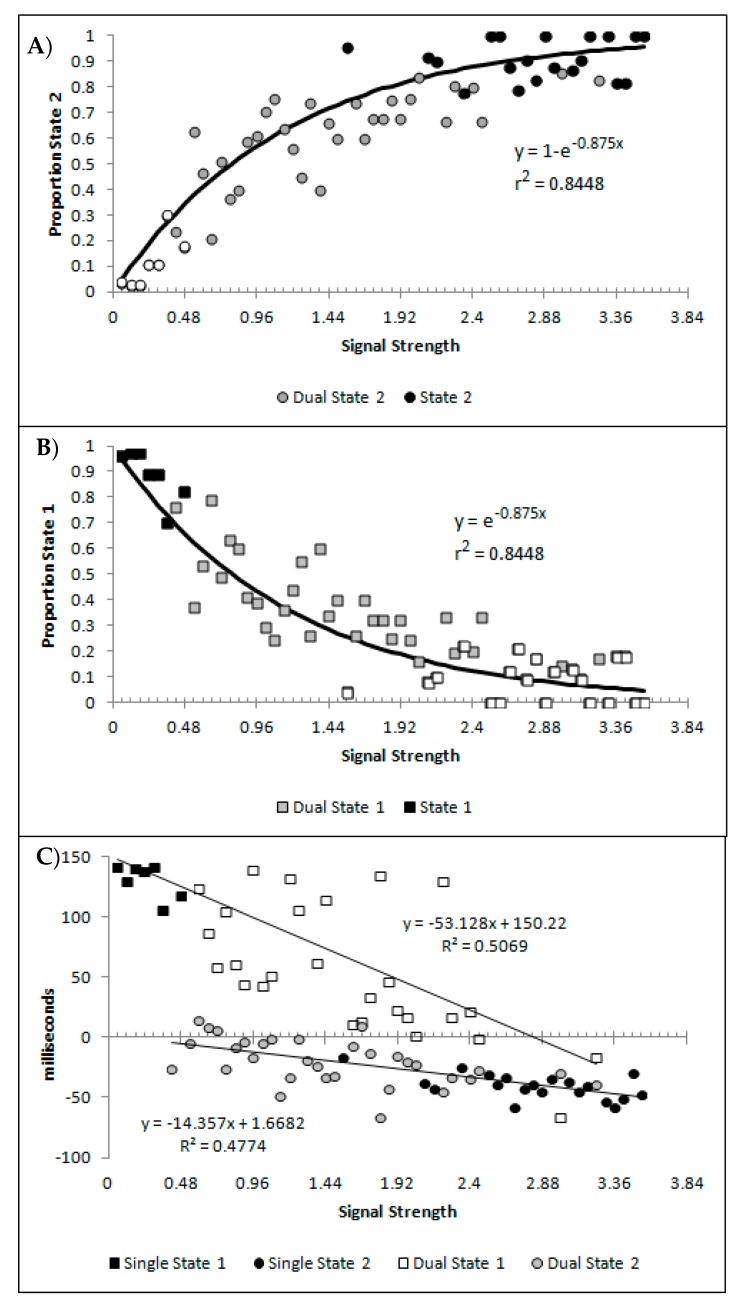
Shown as a function of signal strength, are (**A**) the estimated proportions of state 1 responses (**B**) the estimated proportions of state 2 responses and (**C**) the estimated mean response time of the state 1 and state 2 distributions.

Figure 10A,B show, as a function of the signal strength bins, the percentages of state 1 and state 2 for the blended distribution analysis, regardless of model classification. Symbol fill is used to indicate the bins classified as the corresponding single state (black filled; so by model classification these would all be 100%), blended (grey filled), and the values of the best blend model of the alternative state for bins classified as single state conditions (open symbols; so by model classification these would all be 0%). These are well described by a distance decay function of y = 3.5992 + 1.0097e^−0.875x^, r^2^ = 0.8448. The lower asymptote of 3.5992 was dropped to allow for the theoretically minimum of 0, as was the multiple 1.0097 as this results in a value greater than 1 at signal strengths = 0. The resulting function y = e^−0.875^ also produced r^2^ of 0.8448, and so there was no loss in explanatory power within 4 decimal places. These are consistent with premise 4, that as signal strength increases state 2 responding should become more probable.

Figure 10C shows the mean decision time of the state 1 and state 2 distributions as a function of signal strength. When model selection classified a bin as either a single state 1 or 2, the mean time for the single cross-correlation was selected, and when the bin was classified as dual, the mean for the state 1 and 2 distributions involved in the multiple-cross correlation were selected. The means show a strong linear correlation with signal strength for both state 1 and state 2 estimates (r^2^_(57)_ = 0.5069 and 0.4774, respectively, both *p* < 0.001, pH0|D < 0.001, very strong evidence against the null hypothesis). This is consistent with the expectation that both state 1 and state 2 responding would be faster as the signal strength increased, which is the first aspect of premise 5.

From this analysis, it appears that holding the standard deviations and skews of the state 1 and state 2 distributions, and allowing only their means to vary as a function of stimulus strength, combined with the proportion of responses drawn from the state 1 and state 2 distributions, we should be able to re-create the response time distributions for all 59 signal strength bins. Such a model would contain 10 parameters, a standard deviation and skew for state 1, a standard deviation and skew for state 2, a slope and intercept to predict the mean for state 1, a slope and intercept to predict the mean for state 2, the exponent value 0.875 used to predict the proportion of trials from state 1 (with the proportion from state 2 entirely redundant so not a parameter), and the residual error. Fitting each distribution as per the multiple-cross correlation, while producing the best possible fit, requires 182 parameters (2 standard deviations and 2 skews, 59 individual means for state 1, 59 individual means for state 2, and 59 individual means for the proportion of state 1 trials, plus 1 for error). When fitting the distributions using the model estimates, the fits over the entire 59 signal strength bins were r^2^ = 0.9155. When retaining the 59 best fits from the multiple-cross correlation, r^2^ = 0.9486. This results in BIC values of −5990.69 and −5870.39, respectively, with the lower value indicating that the 10-parameter model is to be preferred. The fits for all 59 distributions can be seen in the Appendix A.

**Figure 11:**
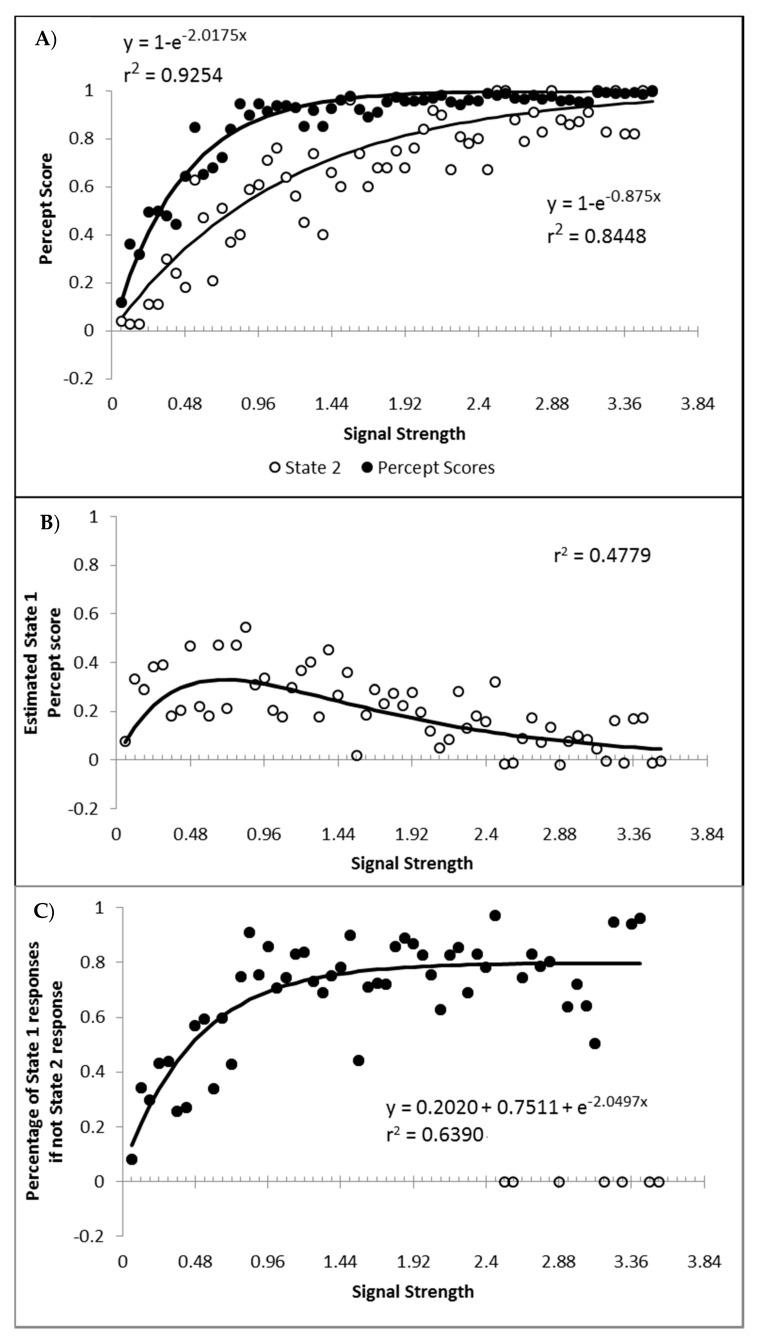
Shown as a function of signal strength, are (**A**) the percept scores (filled symbols) and the estimated proportion of state 2 responses and (**B**) the estimated proportion of total trials requiring state 1 responses, assuming the best possible performance from state 2 responding, (**C**) the estimated proportion of non-state 2 trials requiring state 1 responses, assuming the best possible performance from state 2 responding.

Finally, a question of importance is whether or not responding to the signal arises only from state 2, or are the responses generated to some degree by both state 1 and state 2? If not, then state 1 could be characterised as simply the instances where, for whatever reason, the participant simply did not process the motion signal and generated a random guess. This appears unlikely given that such a formulation would be hard pressed to explain why state 1 responses get faster as signal strength increases. Furthermore, if we hold to be true that state 1 is purely guessing, and allow for state 2 responding to be 100% in accordance to the signal, then the percentage of state 2 trials should at least match the percept scores as the percept scores reflect guess corrected responding. In Figure 11A are displayed the percept scores as actually observed, and the percept scores as calculated if every state 2 trial were in the direction of the stimulus signal. While the percept scores are best described by 1 − a distance decay function y = 1 − (0.0245 + 0.9319e^−2.2084x^), r^2^ = 0.9368, the function y = 1 − e^−2.0175x^, r^2^ = 0.9254 is shown to allow the function to vary between the more theoretically meaningful values of 0 and 1.

In almost every case, the observed percept score is higher than can be explained by the estimated percentage of state 2 responded trials. The difference between the observed percept scores and the percentage of state 2 responding is shown in Figure 11B, as is the corresponding difference between the above fitted functions. The difference between the functions and the observed differences correlates at r^2^ = 0.4779. These values reflect an estimate of the minimum proportion of total trials contributing to the observed percept score arising from state 1 generated responses. When the percentage of state 1 responses is calculated as a percentage of the trials attributed to state 1, and not of the total number of trials, the percentage of state 1 responses corresponding to the signal shows an increase as a function of signal strength that appears to asymptote at approximately 80% (Figure 11C). The conclusion is that responses in the direction of the signal can be generated from both state 1 and state 2, consistent with both premise 1 and 3. Also as expected, the proportion of responses generated by state 1 and state 2 both tend to increase with stimulus strengths.

In summary, a change of state model of consciousness was the basis for an exploratory analysis of the behavioural performance data from a series of _flash_ILM experiments. Starting from the premise that the distribution of response times generated by each state will be stable in shape despite changes in the mean, it was found that the observed distributions of response times for a large range of signals strengths could be explained by a linear decrease in the mean response time for both states combined with a decreasing proportion of responses being generated by state 1. This decrease in the relative proportions followed a distance decay function, with an initial rapid increase in the probability of state 2 responses. The output from the analysis of the response time distributions were used to determine whether it was necessary to suggest that responses in the direction of the signal were being generated by both state 1 and state 2, and this was confirmed. Moreover, the proportion of state 2 responses was found to increase over stimulus intensities, and over the initial range of stimulus intensities the same was found for state 1. State 1, because it appears to generate responses in line with the signal, cannot simply be random guessing. Because state 1 reflects responses generated to signals very close to the subjective point of equality, it is suggested that state 1 would be best described as “non-aware” responding, which is both slow and relatively poorly aligned with the signal. State 2 responding is suggested to reflect “aware” responding, which is fast and much more accurate. The dramatic shift indicative of a change in state is reflected in the large difference between the decision times for state 1 and state 2 responses over all stimulus strengths, particularly where the Bayesian classification analysis indicates state 1 should be considered to contribute; this would correspond to a signal strength in the vicinity of signal strength 2.16. Since the modelled data provides very strong fits to all response time distributions, not only does a change of state model capture the mean decision times and percept scores, it also captures the shape parameters for the distributions as well.

Given that the ILM that arises in this paradigm has been linked with exogenous attention, the inference then is that one of the roles of attention is to increase the effective signal strength of the physical stimulus as this greatly increases the probability of state 2 being activated, which is of particular benefit to very weak signals but also benefits all signals to some extent. State 2 results in a much faster and more signal consistent response, both of which would be beneficial. It is important to note that in natural settings the resulting illusory motion would almost always be consistent with the direction of real motion and the conflict situation where the signal is reduced is an artificial one possible in the lab. Exogenous attention has been shown to both reduce noise and boost signals [52] and both of these properties could account for ILM. The typical attention gradient account for _flash_ILM is based upon a gradient of increased signals. However, noise reduction in the vicinity of the flash would reduce the probability of signals failing to trigger state 2 as the variation in the threshold for triggering state 2 is presumed to reflect background noise levels. A spatial gradient of noise reduction would therefore also produce a spatial gradient of state 2 onset signals that trigger motion detection. This formulation of the exogenous attention explanation for ILM explains the connection between _flash_ILM and the costs plus benefits of exogenous cuing [8] because both arise, at least in part, from the same noise reduction and/or signal amplification properties of exogenous attention.

An alternative way in which attention could trigger ILM is for exogenous attention to have influence in the motion system directly. Viewing a stationary stimulus activates motion detectors in opposite directions with equal strength, as shown by motion after effects such as the waterfall illusion where fatiguing one particular direction of motion detector no longer offsets the influence of the opposite direction. Exogenous attention could amplify motion signals away from the focus of attention, similar to Downing and Treisman [53]’s suggestion that attention biases the interpretation of the display to include motion. The relationship between the costs and benefits from attentional cuing and _flash_ILM would then be capturing an individual’s general “amplification” level associated with exogenous attention. The difference between this formulation and the usual attentional gradient explanation for _flash_ILM is that rather than amplifying the detection of the stimulus onset, which in the case of _flash_ILM creates a series of temporal onsets that then flow on to create a motion signal and in cuing speeds the target to further discrimination processing systems, the amplification for target discriminations and motion are in separate information flows but there is a common magnitude of amplification where ever exogenous attention is applied. This latter suggestion, however, would imply that once the real motion signal is sufficiently different from stationary the heightened response potential of motion detectors near stationary becomes irrelevant as they no longer respond to the input. This would predict that real motion and illusory motion would no longer combine or interfere. While that lack of a combination or interference at slow real motion has not been shown, current studies may not have investigated a sufficient range of real motion to detect this drop off if it does, in fact, even occur. In either case, one of the functions of exogenous attention can be described as increasing the probability of a given physical signal to induce state 2 by increasing the psychological signal strength. When state 2 is induced, decisions are made more quickly (evidenced by the large difference in mean response times between state 1 and state 2 at a constant signal strength), and more consistent with the signal (as evidenced by the percept scores). 

The fact that state 1 responding is far more probable at weak signal strengths, and state 2 responding is far more probable at high signal strengths, makes the suggestion that state 1 and state 2 are candidates for non-conscious and conscious responding viable. The large benefit in response times for state 2 over state 1 responding is consistent with the expectations derived from a melting point type of threshold being crossed and not a glow point type of threshold. This would suggest that whatever the nature of the physical change of state that has occurred is, this change of state is capable of having direct input to decision making and therefore the quale of self-control over our decisions is not a form of self-delusion, misrepresentation, or illusion. 

While it does not address Chalmer’s hard problem of why there are qualia in the first place [45], it does make some testable predictions with regards to isolating the physical change in state that this analysis suggests has occurred. While it remains impossible to classify individual trials as being responded to in state 1 or state 2, the systematic and predictable change in proportions of state 1 and state 2 trials should be detectable in measures of brain functioning, such as EEG measures and/or fMRI measures. Patterns of brain functioning that show a proportional change consistent with the proportions of state 1 and state 2 responding could help to isolate what physical change has occurred when the stimulus signal crosses the threshold to induce the change of state. While Chalmer’s view is that isolating such physical changes can never lead to understanding why qualia emerge [45], this view may be overly pessimistic. It may simply be that without knowing what the physical change in state is, it is impossible to know how it produces qualia. It also could be that qualia emerge as a result of physical interactions which we are currently unable to measure or detect. 

There is, of course, also the possibility that the neuronal change of state that allows for the physical system to self-modify behaviour is only correlated with the physical properties that result in qualia but the change in state is not in and of itself the direct cause of experiential qualia. Should this be the case, it still does not alter the accuracy of the experience of self-volition. What it does open for consideration is the possibility that qualia may be best described in terms of a glow point threshold while the neural mechanism of self-volition may reflect a change in state. This implies that there may be conscious experiential qualia associated with both state 1 and state 2 responding. If this is the case, then the suggestion is that the qualia associated with state 1 would be better described as an experience associated with low confidence, which might be described as “having an impression”, or “a gut feeling”, with the lowest levels reflecting “just guessing” despite being better than chance. State 2, however, may produce qualia associated with high confidence responding. This state of high confidence would reflect a neural state that can then influence the response, which is reflected in the experience of self-volition. Once more, the role of attention remains the same, it serves to increase the signal strength and increase the probability of inducing a high-confident state that activates self-volitional responding.

In closing, the analysis of the current data is presented here simply as an investigation as to the feasibility of a change in state model to account for the data, it is not presented as a test between a change in state model and a “glow point” type model. One might be able to explain the change in response time distributions by holding all parameters apart from the rate of information gain in a Ratcliff diffusion model [54], for example. Even if such a model also accounts for the pattern of percept scores, then we simply are left with two viable hypotheses that can account for the data. What is being presented here is simply a proposed analysis approach based upon what a change in state hypothesis would predict, with the results being entirely consistent with the expectations of that hypothesis, and therefore the hypothesis is considered viable, empirically testable, and worthy of investigation. Even if the alternative hypothesis can also be shown to account for the data, that merely would indicate that, unsurprisingly, there is more work to be done. The analysis approach, based upon a sort of multiple regression based on cross-correlation, is also presented as a first pass proof of concept. We hope that it will be further refined by those who investigate analytical approaches as it may provide a more sensitive test to unravelling mixed distributions where bimodality, for example, may be predicted but the distributions separation is not large enough to make this easily detectable.

## Figures and Tables

**Figure A1:**
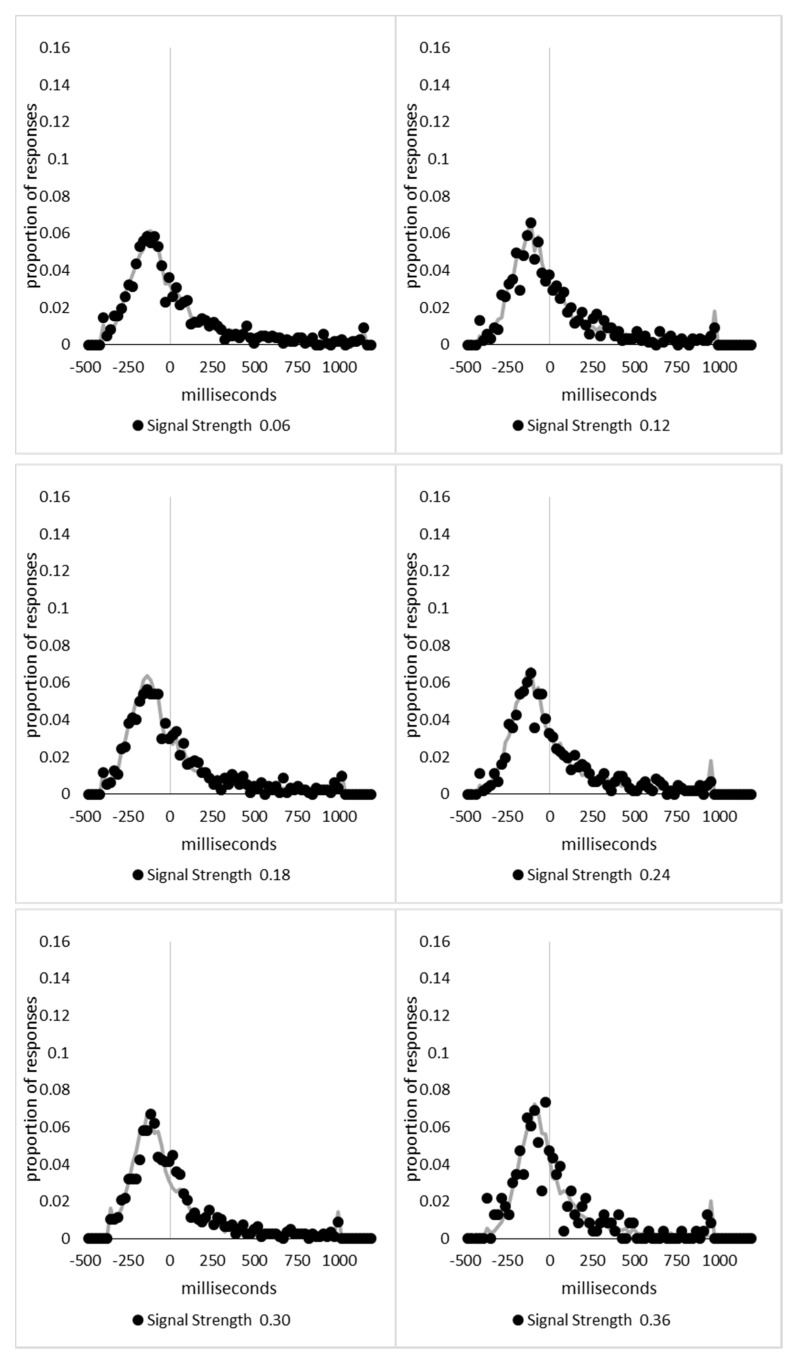
Density functions of the observed data (filled circles) and model (solid line) for signal strength bins 0.06 to 0.36.

**Figure A2:**
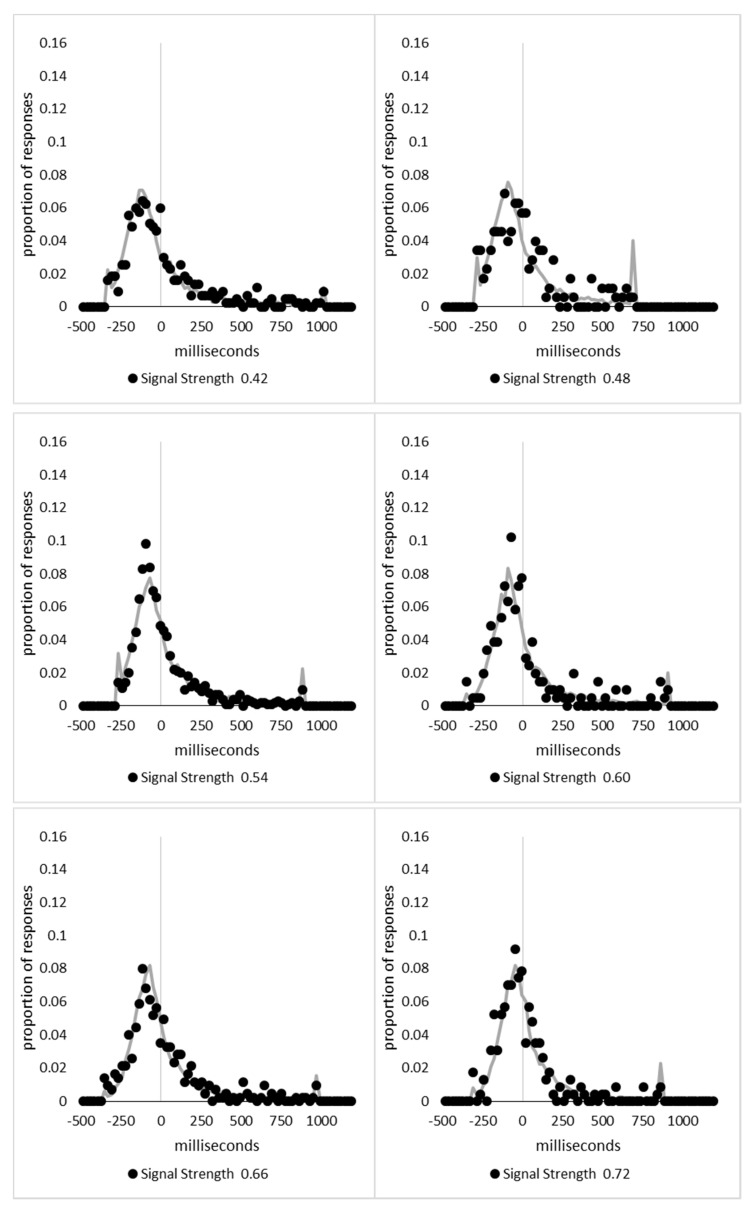
Density functions of the observed data (filled circles) and model (solid line) for signal strength bins 0.42 to 0.72.

**Figure A3:**
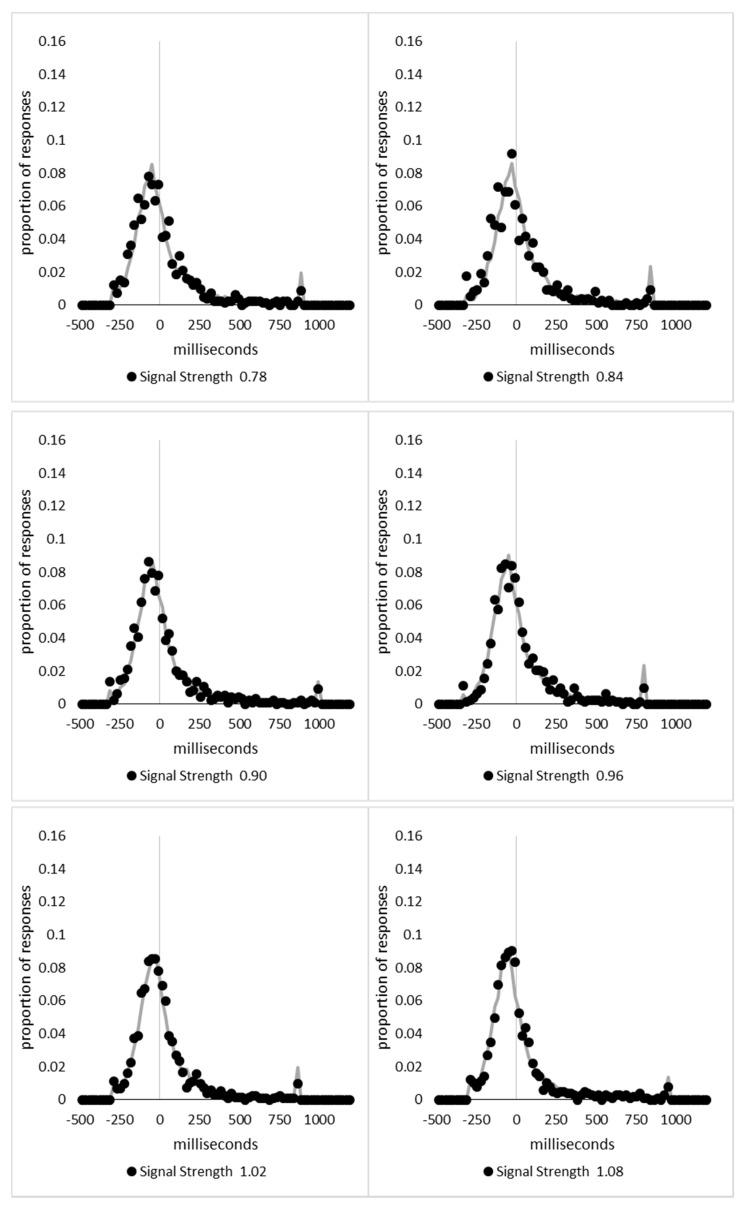
Density functions of the observed data (filled circles) and model (solid line) for signal strength bins 0.78 to 1.08.

**Figure A4:**
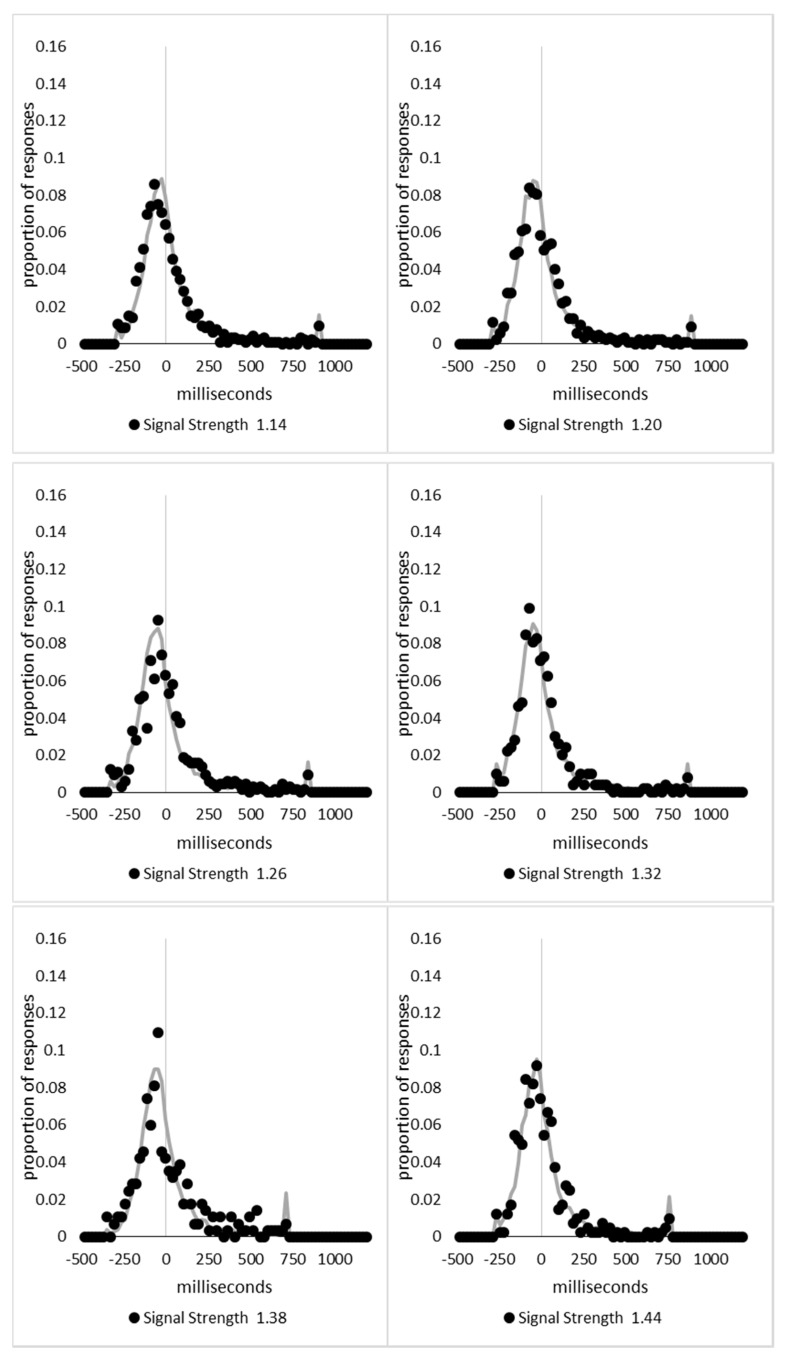
Density functions of the observed data (filled circles) and model (solid line) for signal strength bins 1.14 to 1.44.

**Figure A5:**
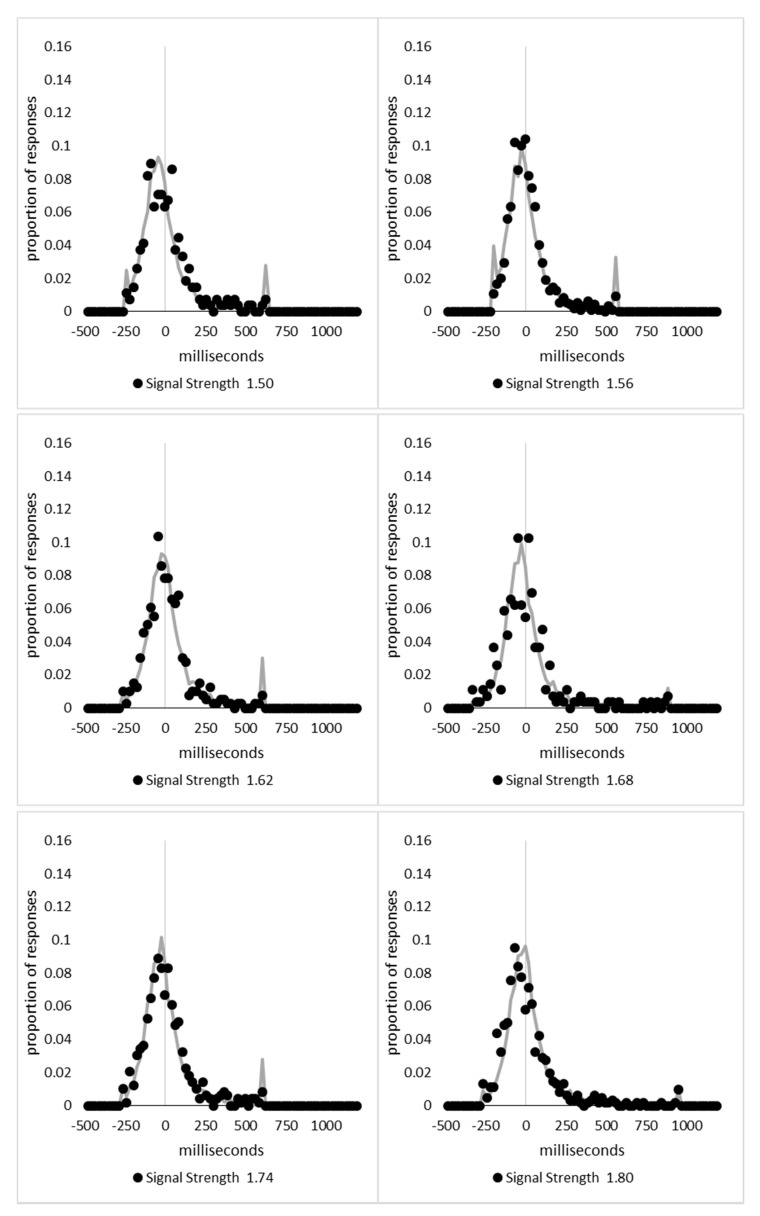
Density functions of the observed data (filled circles) and model (solid line) for signal strength bins 1.50 to 1.80.

**Figure A6:**
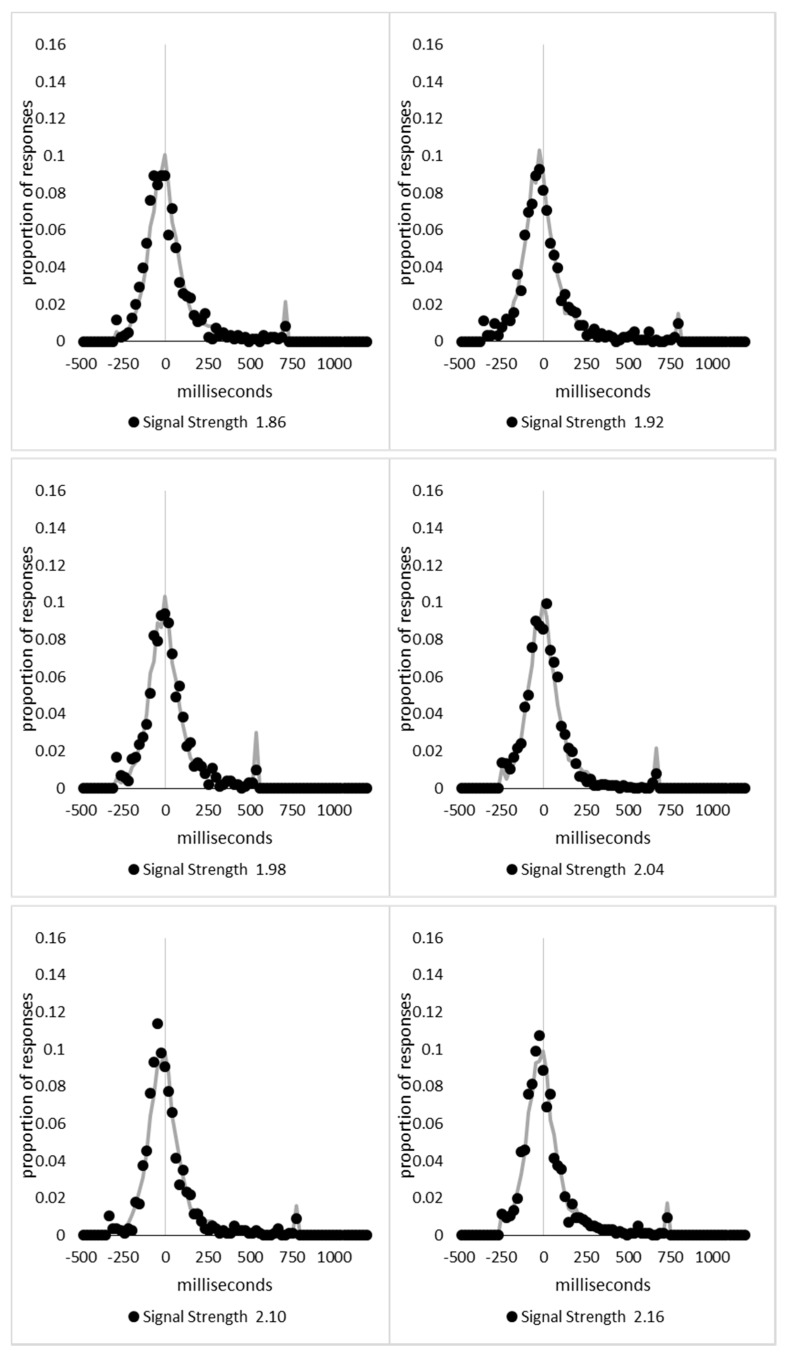
Density functions of the observed data (filled circles) and model (solid line) for signal strength bins 1.86 to 2.16.

**Figure A7:**
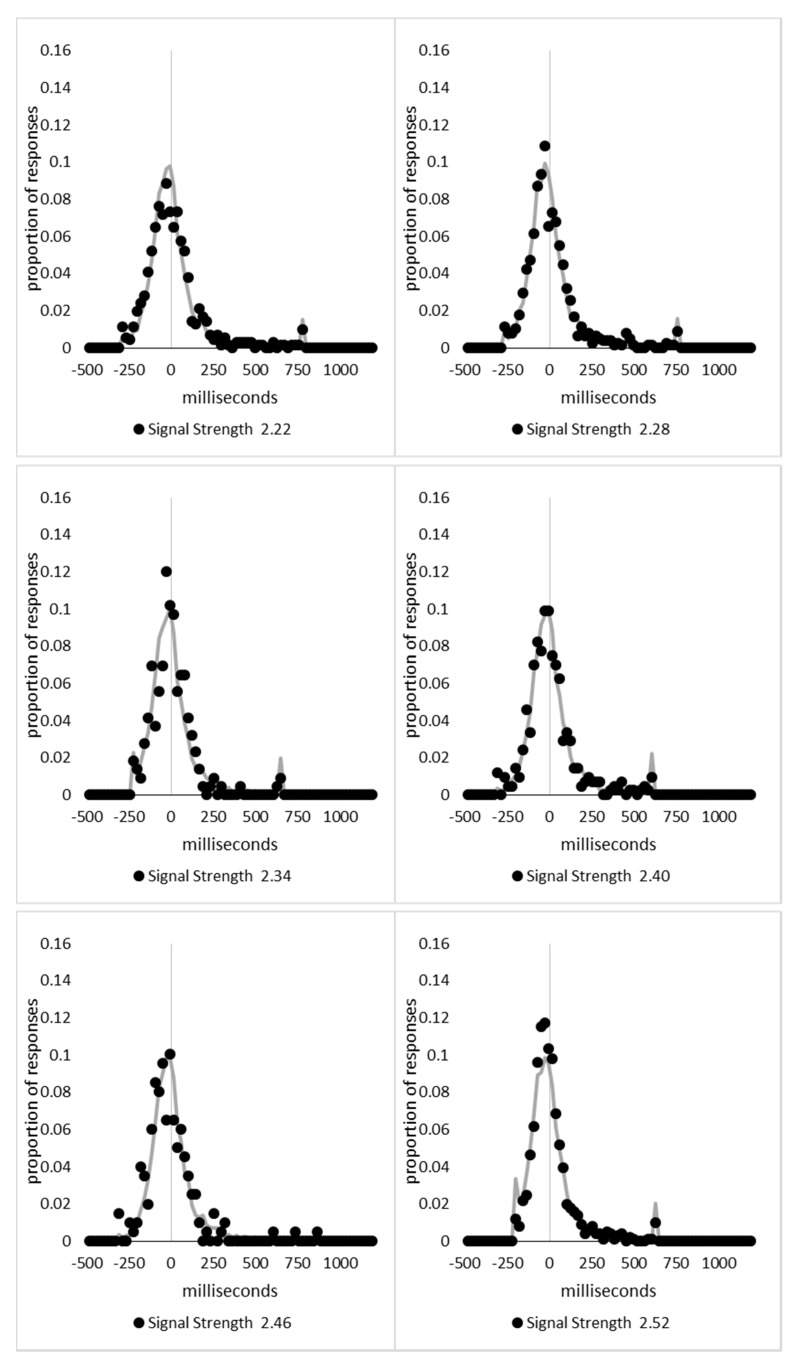
Density functions of the observed data (filled circles) and model (solid line) for signal strength bins 2.22 to 2.52.

**Figure A8:**
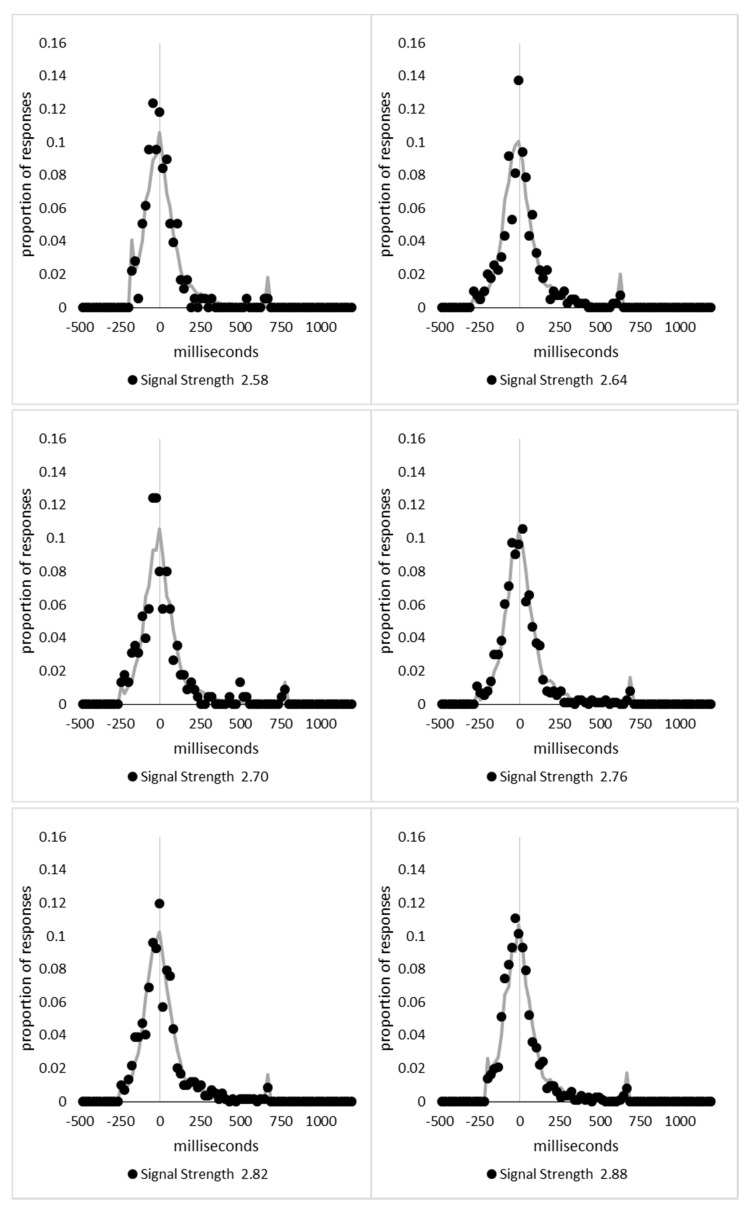
Density functions of the observed data (filled circles) and model (solid line) for signal strength bins 2.58 to 2.88.

**Figure A9:**
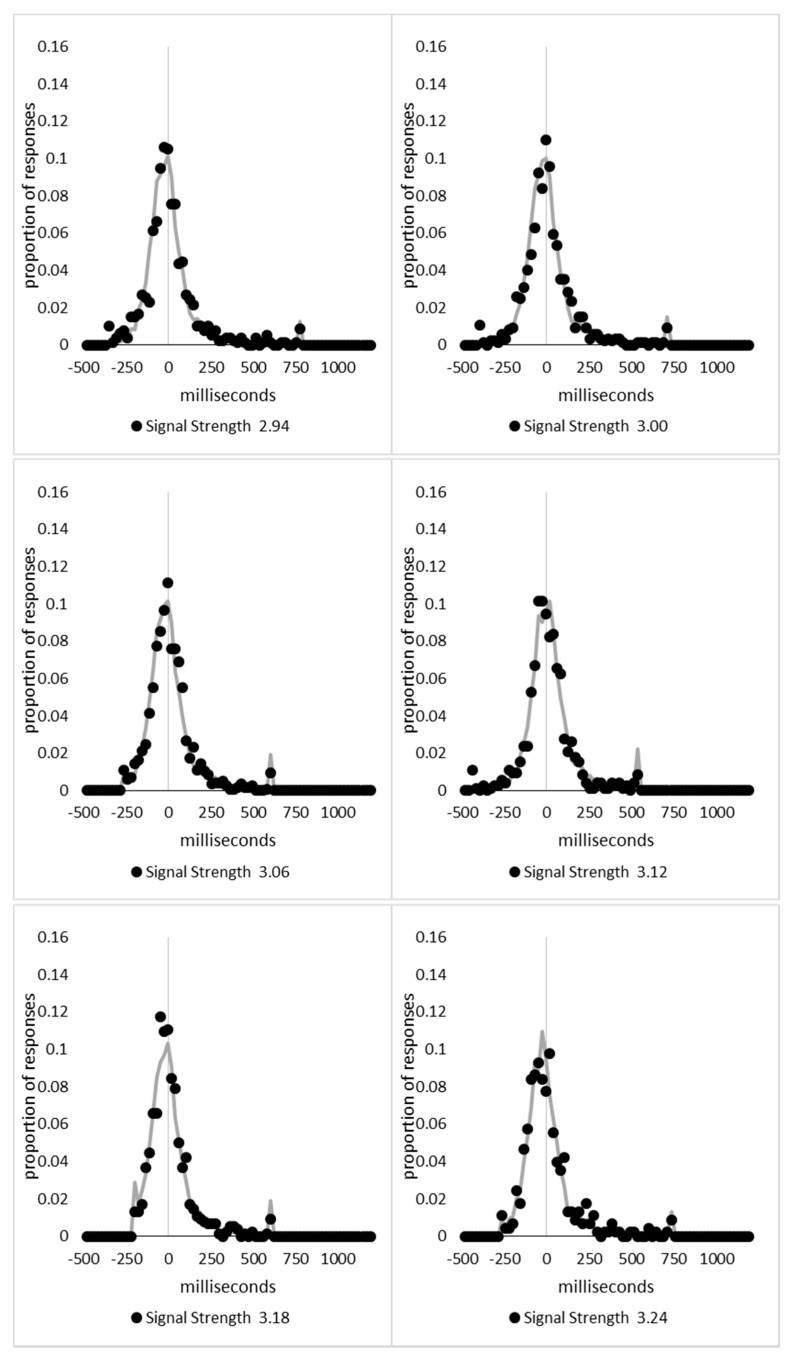
Density functions of the observed data (filled circles) and model (solid line) for signal strength bins 2.94 to 3.24.

**Figure A10:**
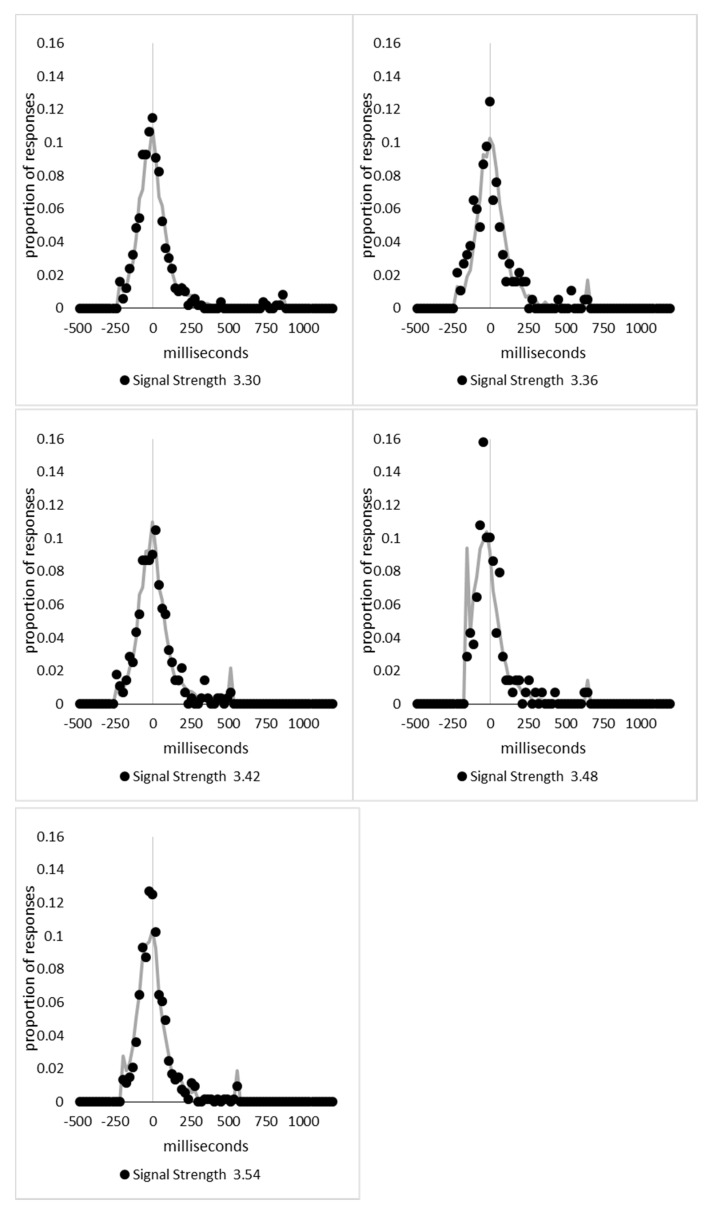
Density functions of the observed data (filled circles) and model (solid line) for signal strength bins 3.30 to 3.54.
